# Non-integrin laminin receptor (LamR) plays a role in axonal outgrowth from chicken DRG via modulating the Akt and Erk signaling

**DOI:** 10.3389/fcell.2024.1433947

**Published:** 2024-07-31

**Authors:** Ewa Mrówczyńska, Karolina Machalica, Antonina Joanna Mazur

**Affiliations:** Department of Cell Pathology, Faculty of Biotechnology, University of Wrocław, Wrocław, Poland

**Keywords:** dorsal root ganglion (DRG), peripheral nervous system (PNS), non-integrin laminin receptor (LamR), laminin, neurite outgrowth, Akt and Erk signaling, chicken embryos, IKVAV peptide

## Abstract

37/67 kDa laminin receptor (LamR)/ribosomal protein SA exhibits dual function as both a ribosomal protein and cell surface receptor for laminin. LamR influences critical cellular processes such as invasion, adhesion, and migration when acting as a receptor. Despite the acknowledged importance of LamR/67LR in various cellular processes, its contribution to the peripheral nervous system development is obscure. Thus, this study investigated the biological activity of LamR in peripheral axonal outgrowth in the presence of laminin-1 or Ile-Lys-Val-Ala-Val (IKVAV) peptide, whose important role in dorsal root ganglia (DRG) axonal outgrowth we recently showed. Unexpectedly, we did not observe LamR on the surface of DRG cells or in a conditioned medium, suggesting its intracellular action in the negative regulation of DRG axonal outgrowth. Using C-terminus LamR-targeting IgG, we demonstrated the role of LamR in that process, which is independent of the presence of Schwann cell precursors (SCPs) and is mediated by extracellular signal-regulated kinase (Erk) and Protein kinase B (Akt1/2/3) signaling pathways. Additionally, we show that the action of LamR towards laminin-1-dependent axonal outgrowth is unmasked only when the activity of integrin β1 is perturbed. We believe that modulation of LamR activity provides the basis for its use for inhibiting axon growth as a potential therapeutic agent for regulating abnormal or excessive neurite growth during neurodevelopmental diseases or pathological nerve regeneration.

## 1 Introduction

The dorsal root ganglia (DRG) are critical peripheral nervous system structures. They have a fundamental role in sensory processing by transmitting sensory information from various receptors (i.e., touch, pain, and temperature) at the periphery to the central nervous system (Nascimento et al., 2018). During early embryogenesis, migrating neural crest cells (NCCs) form DRG, within which they differentiate into peripheral sensory neurons and precursors of glial cells - Schwann cell precursors (SCPs) (Woodhoo and Sommer, 2008). After birth, the mature glial cells - Schwann cells (SCs) play a crucial role in the maturation and myelination of the sensory axons (Hanani, 2005; Jessen et al., 2015).

During peripheral nervous system (PNS) development, neurons, and glial cells navigate through a series of events to establish functional connections with cells of target organs and other neural cells (Song and Poo, 2001). A fundamental aspect of this process involves the dynamic interaction between cellular components and the extracellular matrix (ECM) (Hynes and Zhao, 2000). Cues coming from ECM regulate critical cellular functions such as cell adhesion, migration, and differentiation. The ECM-cell receptor interactions activate these intracellular signaling pathways and influence the reorganization of cellular structures. It has been shown that laminin-1, one of the ECM proteins, is indispensable for PNS development (Desban and Duband, 1997; Masuda et al., 2007). The important role of laminin in embryonic development is evidenced by the fact that laminin is produced already at the four-cell morula stage as the first of the ECM proteins (Shim et al., 1996). Recently, we also demonstrated the crucial role of laminin-1 in the formation of chicken DRG, axonal outgrowth of sensory neurons, and SCPs migratory properties through the action of active site-peptide IKVAV (Mrówczyńska and Mazur, 2021).

One of the players in the intricate molecular dialogue between cells and ECM is the non-integrin laminin receptor (37/67-kDa laminin receptor; LamR; ribosomal protein SA; RPSA). It is a significant mediator in various cellular processes, such as cell adhesion, migration, proliferation, and differentiation (Digiacomo and Meruelo, 2016). All these processes are crucial for proper PNS development, making LamR a potential participant in this process (Donaldson et al., 2000; Fujimura et al., 2005; Wu et al., 2016). LamR apparently binds laminin-1 with high affinity (Wewer et al., 1986). For this reason, the action of this non-integrin receptor is often compared to the action of integrins, which are major mediators of cell-ECM interactions, including laminins (Heino and Kapyla, 2009).

The presence of a transcript *RPSA* has been reported in the DRG of the chick embryo, whereas the presence of laminin-1 was observed in the mesenchymal tissues surrounding these structures (Masuda et al., 2009). The role of laminin-integrin signaling in neuronal growth is thoroughly studied, while the involvement of LamR as a laminin-binding protein in PNS formation remains poorly understood. This inspired us to delve into the molecular and cellular aspects of LamR’s role in this process to understand the intricate interplay between ECM receptors and neuronal elements in regulating peripheral axonal growth.

Anomalies or dysfunctions in DRG development can lead to sensory disorders and chronic pain conditions such as Congenital Insensitivity to Pain (CIPA), sensory neuropathy, and neuropathic pain. Up to now, the application of anti-LamR antibodies to interrupt its functionality is a commonly studied *in vitro* and *in vivo* approach in potential therapies, e.g., cancers, neuroblastoma, or Alzheimer’s disease (Chetty et al., 2014; Rebelo et al., 2016; Vania et al., 2016; Ferreira et al., 2018). Therefore, we utilized monoclonal anti-LamR antibodies to understand the role and mechanisms of LamR action in the proper development of PNS based on DRG. This may hold significance for clinical applications targeted at LamR for sensory disorders, opening the door to potential therapeutic interventions.

## 2 Materials and methods

### 2.1 Culture conditions of DF-1 cell line

Chicken embryonic fibroblast DF-1 (UMNSAH/DF-1 - CRL-12203) were purchased from ATCC^®^ (American Type Culture Collection) and cultured in Dulbecco’s modified Eagle medium (DMEM) (ATCC^®^), supplemented with 10% fetal bovine serum (FBS) in a humidified atmosphere at 39°C and with 5% CO_2_ accordingly to ATCC^®^ recommendations.

### 2.2 Primary cell culture-isolation and culture of DRG

DRG were isolated from the trunk area of an 8-day-old (E8) chick embryo’s neural tube using previously published methods (Kuhn, 2003; Powell et al., 2014). For the single-cell culture of sensory neurons and SCPs, DRG were enzymatically dissociated by gentle trituration using a 20–200 µL pipette and trypsinization (Kuhn, 2003). DRG were maintained in complete DRG culture medium [98% DMEM/F-12 medium (Thermo Fisher Scientific), 0.5 mg/mL bovine serum albumin (Merck), 2 mM L-glutamine (Thermo Fisher), 1x N-2 supplement (Thermo Fisher Scientific), 100 ng/mL nerve growth factor (NGF) (Merck)] (Kuhn, 2003). DRG cells or DRG explants were cultured on glass coverslips coated with poly-D-lysine (PDL) (0.1 mg/mL) (Santa Cruz Biotechnology) for 1 h at RT, followed by washing with sterile water and drying. DRG were cultivated for 48 h at 37°C with 5% CO_2_. Additionally, laminin-1 from Engelbreth-Holm-Swarm murine sarcoma basement membrane (laminin-1) (Sigma-Aldrich) (10 μg/mL) or MAPTRIX-L-IKVAV (20 μg/mL) (Sigma-Aldrich) were added to the medium. To inhibit the proliferation of SCPs in DRG culture, deoxycytidine analog - 1-β-D-Arabinofuranosylcytosine (Ara-C) (Sigma-Aldrich) was added to the culture in the concentration of 0.24 μg/mL (Kuhn, 2003).

### 2.3 Antibodies-mediated disruption of protein function

To disrupt LamR activity, monoclonal mouse anti-Laminin-R Antibody (anti-LamR_254-290_ IgG) (Santa Cruz Biotechnology; clone A-7; sc-376295) or rabbit polyclonal RPSA Antibody (anty-(LamR_218-230_ IgG) (Thermo Fisher Scientific; PA5-86634) were added to the medium at a dose of 10 μg/mL. Under control conditions, DRG were cultured with identical amounts of control antibodies from non-immunized mice–normal mouse IgG (Santa Cruz Biotechnology; sc-2025) or normal rabbit IgG (Cell Signaling Technology; #2729). To inhibit or activate integrin β1, 10 μg/mL of anti-integrin β1 antibodies CSAT (Developmental Studies Hybridoma Bank; CSAT) or TASC/9D11 (Sigma-Aldrich; MAB19294) were used, respectively. IgG were added to the medium at day 0 of the cultivation of isolated DRG. DRG were cultured for 2 days (48 h) and then proceeded to analyses.

### 2.4 Immunocytochemistry and confocal microscopy

Both permeabilized and non-permeabilized conditions were used to investigate the cellular localization of proteins. For immunocytochemical staining in permeabilized conditions, DRG grown on glass coverslips coated with PDL were fixed with 4% formaldehyde (FA) solution (paraformaldehyde purchased from Sigma-Aldrich) for 20 min before being permeabilized with 0.1% Triton X-100 (Sigma-Aldrich) in phosphate-buffered saline (PBS) by 6 min incubation at RT. A blocking solution [1% BSA (Sigma-Aldrich) and 0.1% Triton X-100 (Sigma-Aldrich) in PBS] was used to avoid nonspecific bindings of antibodies. The coverslips were incubated with primary antibodies that were diluted in a blocking solution overnight at 4°C. The coverslips were then washed with PBS and incubated with secondary antibodies conjugated with Alexa-Fluor dyes. When required, Hoechst 33342 (Thermo Fisher Scientific; 1:1,000) was used to stain the nuclei, and phalloidin conjugated with a fluorescent dye (Santa Cruz Biotechnology; 1:100) to stain filamentous actin (F-actin). Anti-LamR (Santa Cruz Biotechnology; A-7; sc-376295; 1:50), anti-LamR (Thermo Fisher Scientific; PA5-86634; 1:100), anti-Sox10 (Santa Cruz Biotechnology; N-20; sc-17342; 1:50), anti-NF-m (DSHB; 4H6, 1:6), anti-laminin-1 (Sigma-Aldrich, L9393; 1:500), and anti-GFP (Proteintech; 50430-2-AP; 1:200) antibodies were used for immunocytochemistry (ICC). After an additional round of washings, coverslips were mounted on glass slides with Dako Mounting Medium (Agilent Technologies).

For non-permeabilized conditions, the cell culture medium was removed, and coverslips were immediately incubated with primary antibodies and phalloidin (diluted in 1% BSA in PBS) for 20 min at 4°C. Since phalloidin binds to F-actin, which localizes intracellularly, the absence of a signal for F-actin indicated that non-permeabilized conditions were maintained during the procedure. Positive control was detecting extracellular fragment of β1 integrin using antibodies directed against it. Subsequently, coverslips were washed thrice with PBS, and fixed with 4% FA for 20 min at 4°C. The cell fixation step was performed after incubation with primary antibodies because formaldehyde can affect the integrity of the cell membrane, thus allowing antibodies or bigger fluorescent dye to enter the cell (Cheng et al., 2019). After fixation, coverslips were processed as described for permeabilized conditions.

For ICC-negative controls (nonspecific fluorescent signal of secondary antibodies), samples were incubated only with secondary antibodies, and the incubation with primary antibodies was omitted. Detection of proteins and data analysis (histograms of fluorescence signals) were done using Leica TCS SP8 Confocal Laser Scanning Microscope with Leica Application Suite X (LAS X) software. Each color channel was usually presented in a grayscale.

### 2.5 Internalization of anti-LamR IgG

To evaluate whether antibodies added to the medium for disruption of protein’s function could be internalized by DRG cells, DRG were cultured on PDL-coated surface, supplemented with IKVAV or laminin-1, and additionally treated with control mouse IgG (Santa Cruz Biotechnology; sc-2025), or anti-LamR IgG (Santa Cruz Biotechnology; A-7; sc-376295). After 48 h DRG were fixed and preceded for ICC in permeabilized conditions. However, we omitted the step of incubation with the primary antibodies. We incubated microscopic slides with goat anti-mouse secondary antibodies (Thermo Fisher Scientific; A-11029; 1:400) to detect IgG added to the culture. Photos of DRG for this experiment were taken using the same settings (exposure time, gain, etc.).

### 2.6 Neurite outgrowth assay

Axonal outgrowth has been assessed according to the neurofilament medium chain (NF-m) detection, as a marker for neurites. The *Fiji* application with a semi-automated NeuronJ plugin was chosen for tracking individual neurites (Meijering et al., 2004; Schindelin et al., 2009). The length of ten of the most extended neurites per DRG was measured, and values were then averaged. Usually, nine DRG for each condition were examined in at least three independently conducted tests.

### 2.7 Indirect adhesion assay adapted from XTT viability assay

DRG were dissociated by gentle trituration followed by 20 min incubation in a 0.25% trypsin/0.05% EDTA solution to obtain the single-cell culture of sensory neurons and SCPs. 30,000 cells were seeded into wells of a 96-well plate covered with PDL. Cells were seeded in a standard DRG medium with supplementation of IKVAV or laminin-1. 30 min before seeding, control IgG or anti-LamR_254-290_ IgG were added to the suspension of DRG cells. Plates were incubated at 37°C with 5% CO_2_ for 3 h to allow cells to adhere. To indirectly measure the adhesion abilities of cells, a CyQUANT™ XTT Cell Viability Assay (Thermo-Fisher Scientific) assay was adapted and that metabolic activity of cells correlates with the portion of well-adhered cells. Three technical repeats for each condition were performed and then values were averaged for each test. Three independent assays have been performed. Absorbance values were normalized to the average values of the control group - control IgG.

### 2.8 Elimination of SCPs and evaluation of the impact of anti-LamR IgG on axonal outgrowth

To inhibit the proliferation of SCPs in DRG culture, we used Ara-C (Sigma-Aldrich), which was added to the culture medium simultaneously with seeding DRG on day 0. After 48 h, DRG were fixed and taken for ICC analyses. The number of SCPs was determined based on anti-SOX10 staining (a marker for SCPs). The “Particle analysis” function in Fiji application with manually operated thresholds corresponding to the SOX10+ cells visible beyond the DRG was applied for the estimation of the total number of SCPs. For the statistical analysis, nine DRG were analyzed per each condition. Four images were taken corresponding to the four-quarters of DRG. The number of SCPs was counted from each image and the average per one DRG was assessed. Usually, nine DRG for each condition were examined in three separate experiments.

### 2.9 DRG samples preparation and Western blot analysis

The preparation of cell lysates and Western blot has been done as described elsewhere with slight modifications (Makowiecka et al., 2016; Mazur et al., 2016)*.* Briefly*,* DRG were lysed by scraping them in cytoskeletal-bound protein extraction buffer (10 mM Tris-HCl pH 7.4, 100 mM NaCl, 1 mM EDTA, 1 mM EGTA, 1 mM NaF, 20 mM Na_4_P_2_O_7_, 2 mM Na_3_VO_4_, 1% Triton X-100, 10% glycerol, 0.1% SDS, 0.5% sodium deoxycholate) with the addition of protease inhibitor cocktails 2 and 3 (Sigma-Aldrich). Next, the samples went through three freeze-thaw cycles and then were centrifuged at 12,000 x *g* for 5 min at 4°C. Collected supernatants were stored at −80°C. For conditional media samples, media were collected and concentrated using the *Amicon Ultra-0.5 Centrifugal Filter Unit* (Sigma-Aldrich). The protein concentration within the samples was determined using Bradford protein assay (Sigma-Aldrich) or the Pierce BCA Protein Assay Kit (Thermo Fisher Scientific), according to the manufacturers’ protocols. The samples for SDS-PAGE were prepared using the 4 × Sample Loading Buffer (40% glycerol, 240 mM Tris–HCl pH 6.8, 8% SDS, 0.04% bromophenol blue, 5% β-mercaptoethanol). SDS-PAGE separated proteins holding 7.5–30 µg of total protein were then transferred onto nitrocellulose membranes. 5% skim milk or 5% BSA dissolved in TBS-T buffer (20 mM Tris, 150 mM NaCl, 0.1% Tween-20) was applied to block the membranes. The mass marker was a PageRuler Prestained Protein Ladder (Thermo Fisher Scientific). Primary and secondary antibodies, which are listed in Supplementary Table S1 were diluted in the blocking solution. The immunoblots were developed using the Clarity Western ECL Substrate (Bio-Rad). The ChemiDoc MP System and Image Lab 4.0 software (Bio-Rad) were applied to detect chemiluminescence emission in the immunoblots, analysis of the results, and densitometry. The intensity of bands was standardized to whole protein content (determined using Ponceau S staining) and GAPDH level (if applicable), and then normalized to the control groups. 20–30 DRG have been harvested for each sample. Three sets of lysates from separate DRG cultures and experiments have been examined for densitometric analyses.

### 2.10 DNA constructs and transfections of DF-1 cell line

A vector for the *LamR* expression was prepared based on pCIG-V5-Snail2-IRES-nls-GFP plasmid, which was a gift from Martin Cheung (Addgene plasmid #44282) (Liu et al., 2013). Snail2 coding sequence was removed using *Cla*I and *Xho*I restriction enzymes (Thermo Fisher Scientific). Afterward, the pCIG-V5-IRES-nls-GFP vector was isolated from a 0.5% agarose gel in TAE with GenElute™ Gel Extraction Kit (Sigma-Aldrich) and recircularized with T4 DNA ligase (Thermo Fisher Scientific). The sequence coding for LamR was amplified using Phusion™ High-Fidelity DNA Polymerase (Thermo Fisher Scientific). cDNA from E3.5 chicken embryo acted as a template. RPSA_fwd: TCA​TTT​TGG​CAA​AGA​ATT​GCT​CGA​GGC​GCC​ACC​ATG​TCC​GGA​GG and RPSA_rev gaa​ttc​gat​atc​aag​ctt​at*cga​tcc*TTA​AGA​CCA​CTC​CGT​GGT​AGT​CC primers have been used. The verified PCR product was cloned into pCIG-V5-IRES-nls-GFP plasmid in the NEBuilder HiFi DNA Assembly Reaction (New England BioLabs). Simultaneously, control plasmid pCIG-V5-IRES-nls-GFP was prepared. For this purpose, the pCIG-V5-IRES-nls-GFP vector isolated from a 0.5% agarose gel using GenElute™ Gel Extraction Kit (Sigma-Aldrich) has been purified using GenElute Gel Extraction Kit (Sigma-Aldrich), treated with polymerase T4 to rebuild restriction site, and then recircularized with T4 DNA ligase (Thermo Fisher Scientific). Verified by sequencing (Microsynth AG), proper plasmids were purified using an endotoxin-free plasmid DNA isolation kit - NucleoBond Xtra Midi Plus EF (Macherey-Nagel).

30,000 DF-1 cells were seeded on 12 mm round coverslips for cell transfection. 48 h later, according to the manufacturer’s recommendations, they were transfected with a plasmid coding for LamR or control plasmid with the help of Lipofectamine 3,000 reagent (Thermo Fisher Scientific). 24 h later, the cells were fixed with 4% FA in PBS for 20 min at RT and subjected to immunocytochemical staining. The used construct allowed for the separate production of GFP and LamR, as they did not form LamR-GFP fusion protein.

### 2.11 Statistical analysis

Alignment of amino acid sequence of chicken and human LamR was done using Clustal Omega - Multiple Sequence Alignment (EMBL’s European Bioinformatics Institute (EMBL-EBI). Figure 8 was modified with text, markings (stars), and annotation after the adaptation of “Stomach” from Servicer Medical Art by Servier, licensed under a Creative Commons Attribution 3.0 Unported License”. GraphPad Prism 7 and 8 (GraphPad Software) were used to prepare statistical analyses and generate graphs. First, the Shapiro-Wilks test, D'Agostino, and Pearson’s tests were applied to determine whether the distribution was normal. Parametric and nonparametric versions of tests for normal and abnormally distributed data sets were performed, respectively. Depending on the data sets and conditions, statistical significances were determined by applying the two-tailed unpaired Student’s t-test or an ANOVA with posthoc tests. Data on graphs were presented as a mean ± SD. The significance levels were set as *p* ≥ 0.05 (non-significant; ns); *p* < 0.05 (*), *p* < 0.01 (**), *p* < 0.001 (***), and *p* < 0.0001 (****).

### 2.12 Animals and ethics statement

Based on the Polish and European Acts on the Protection of Animals Used for Scientific or Educational Purposes, performing the experiments using chicken (*Gallus gallus domesticus*) embryos, classified as HH34 (E8) stage by Hamburger and Hamilton, did not require formal permissions. To minimize any potential pain or suffering, as few chicken embryos as feasible were used for each experiment. To obtain a chicken embryo at stage HH34 (E8), fertilized chicken eggs were incubated at 37°C and 80% humidity for 8 days.

## 3 Results

### 3.1 Intracellular localization of LamR in DRG

**FIGURE 1:**
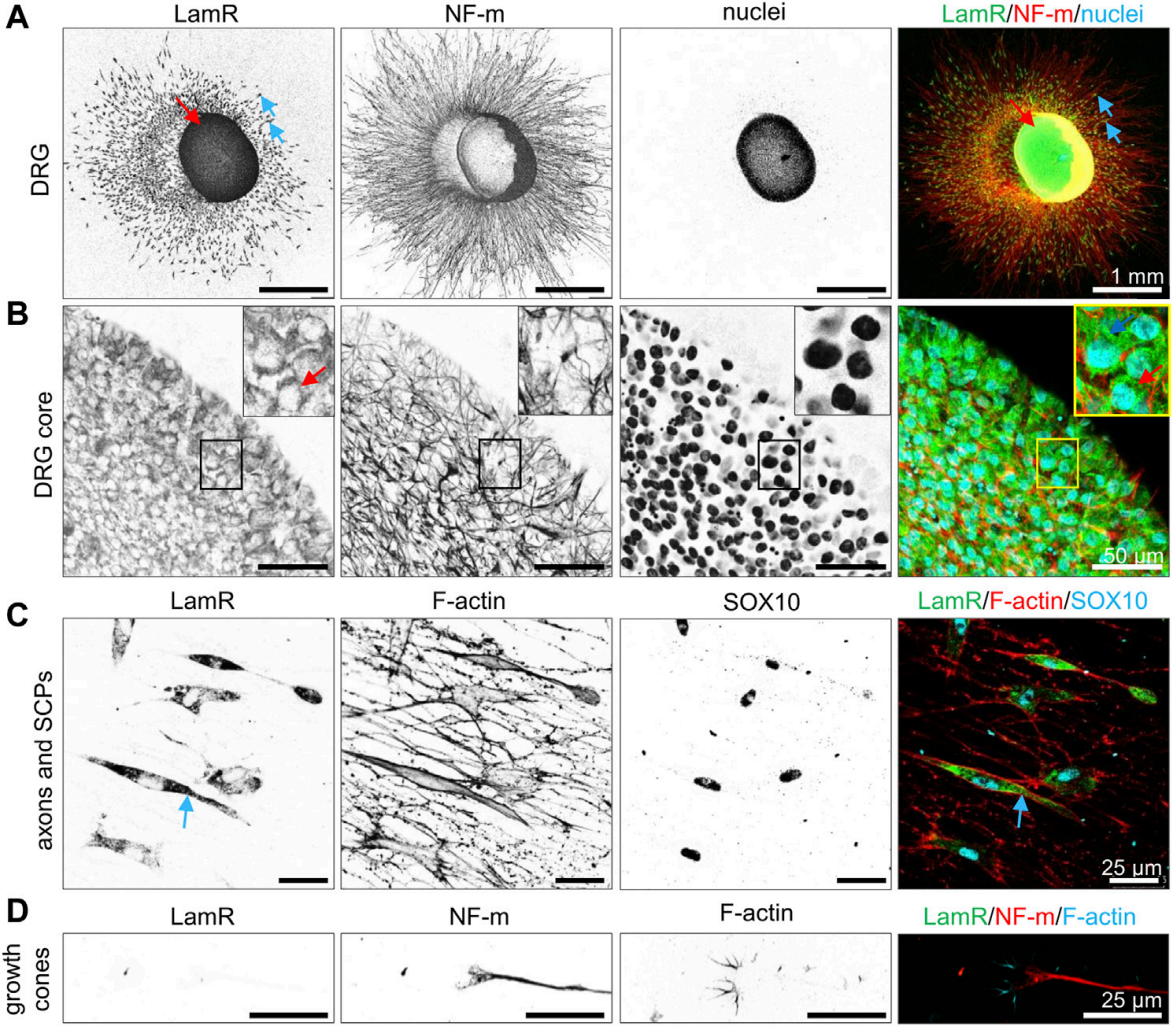
Localization of LamR’s in cells of DRG. DRG cultured for 48 h were immunostained under the cell membrane permeabilizing conditions. **(A, B)** DRG stained with nuclei-specific dye (Hoechst 33342), anti-NF-m (neurites), and anti-LamR_254-290_ antibodies. **(B)** A magnified view of the cell bodies. **(C)** SOX10-expressing SCPs with underlying axons. Fluorescently conjugated phalloidin was used to detect F-actin. **(D)** Growth cones displaying F-actin-rich filopodia (phalloidin-Alexa Fluor™ 568) and NF-m were detected in the growth cone and axons. Blue arrows indicate SPCs, while red arrows indicate the somas of sensory neurons. SCPs, Schwann cell precursors.

**FIGURE 2:**
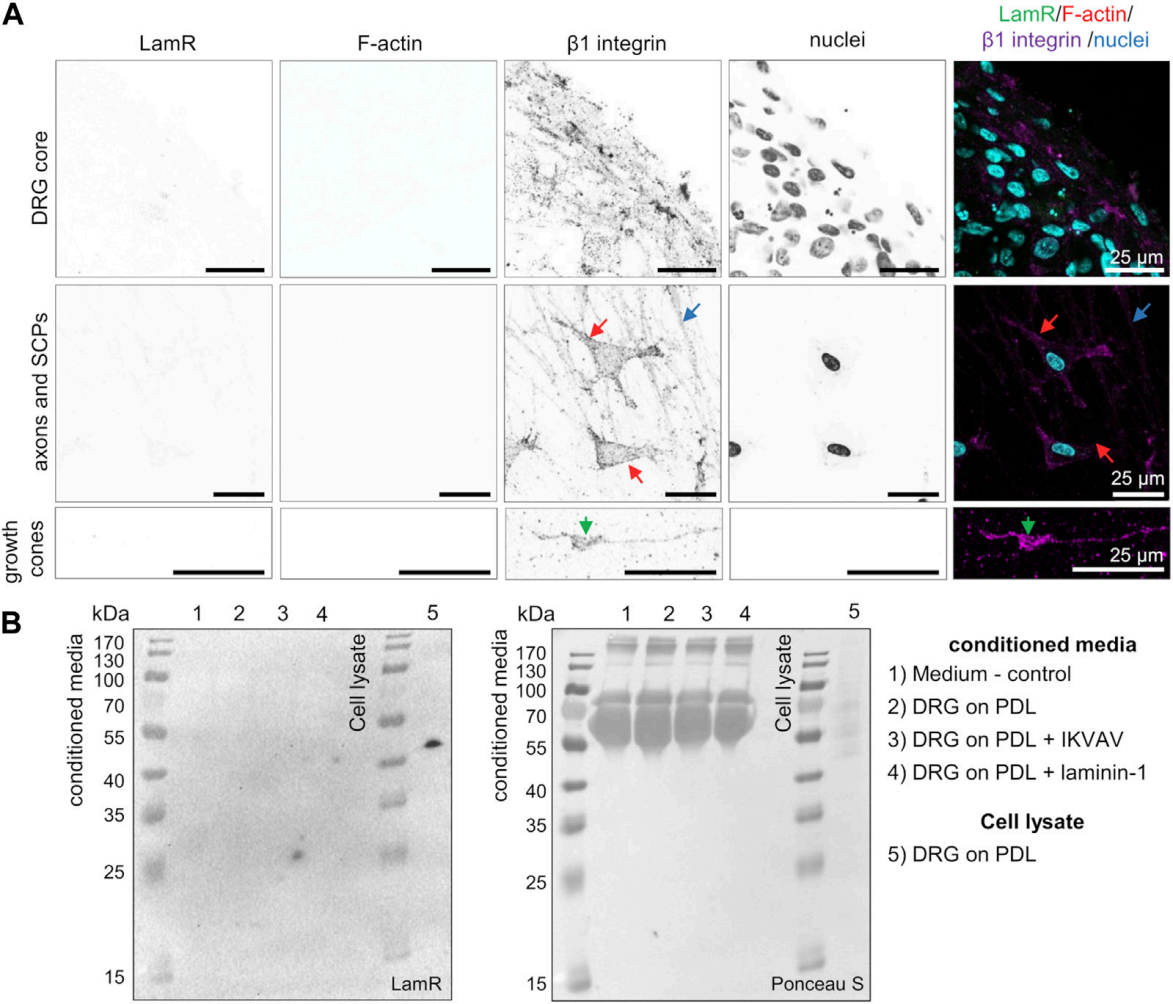
LamR is absent on the surface of DRG cells and in a conditioned medium. **(A)** DRG were subjected to staining under cell-non-permeabilizing conditions. Antibodies directed against LamR_254-290_ or β1 integrin, together with phalloidin Alexa Fluor™ 350 (F-actin) and Hoechst 33342 dye (cell nuclei), were used to stain the fixed DRG. A positive β1 integrin signal and negative signal for F-actin revealed that solely cell surface proteins were detected. The panels show a magnified view of sequentially DRG, axons and SCPs, and growth cones. SCPs are pointed by red arrows, axons by blue, and growth cones by green ones. **(B)** Evaluation of LamR secretion by DRG cells growing in studied conditions. Conditioned media were evaluated for LamR detection using the Western blot technique. Ponceau S-stained membrane acted as a loading control. PDL, poly-D-lysine; SCPs, Schwann cell precursors.

Before studying the action of LamR on DRG axonal outgrowth, we first investigated LamR localization in DRG. Using ICC, we verified comprehensively where LamR was located in embryonic DRG cells. ICC analyses in permeabilized conditions revealed cytoplasmic localization of LamR in somas of sensory neurons (Figures 1A, B). However, we observed the absence of LamR in the axons of sensory neurons (Figures 1A, C, D). Furthermore, LamR was absent in the growth cones of sensory neurons, which were identified using a combination of NF-m and F-actin detection (Figure 1D). On the other hand, LamR was found in the cytoplasm of SCPs identified as cells expressing SOX10 (Figure 1C). Since we considered LamR as a laminin receptor, we verified whether LamR was present on the cell membrane. For this purpose, we conducted immunostaining in non-permeabilized conditions. To ensure we targeted LamR exclusively on the cell membrane, we performed a two-step control analysis of non-permeabilized conditions. The positive control was the detection of β1 integrin’s extracellular motif (Figure 2A). The negative control was the detection of F-actin, which is exclusively present intracellularly (Figure 2A). We found that LamR was absent on the outer side of the plasma membrane of sensory neurons or SCPs (Figure 2A). Negative controls for ICC analyses (permeabilized and non-permeabilized conditions) are presented in Supplementary Figure S1. The secretion of LamR to the culture medium could allow its interaction with any ligand like laminin-1. To investigate the other possibility for LamR’s potential interactivity with the extracellular ligands, we verified whether LamR was secreted into the medium by DRG cells. Western blot analysis of media lysates of DRG in the presence of poly-D-lysine (PDL), laminin-1, or IKVAV (conditions used in following experiments) showed the absence of LamR in a conditioned medium, indicating that LamR was not secreted by DRG cells under studied conditions (Figure 2B). Staining the immunoblots with Ponceau-S functioned as a loading control. LamR signal detected on DRG cell lysates acted as a positive control (Figure 2B).

The findings revealed intracellular localization of LamR in somas of sensory neurons and SCPs. Despite the known role of LamR as the receptor for laminin-1, LamR was not present on the outer side of the plasma membrane and was not secreted to the medium.

### 3.2 Impact of LamR on laminin-1 and IKVAV-dependent DRG axonal outgrowth in the presence and absence of SCPs

Laminin-1 has multiple cell receptor binding sites, influencing cell migration, differentiation, and neuronal outgrowth (Kleinman et al., 1990). One of them - pentapeptide IKVAV, is known to promote cell attachment and migration and, most importantly - neurite outgrowth (Tashiro et al., 1989; Dhoot et al., 2004). We recently confirmed that laminin-1 (independent of the treatment way) and IKVAV stimulate axonal outgrowth, particularly in chicken embryonic DRG (Mrówczyńska and Mazur, 2021). In this paper, we decided to investigate whether LamR, the potential receptor for laminins, plays a role in laminin-1- and IKVAV-dependent stimulation of axonal outgrowth in chicken embryonic DRG.

*RPSA* product can function as a ribosomal protein, so affecting the expression level of *RPSA* would cause serious consequences for cell viability, causing cell death, as shown previously (Kaneda et al., 1998; Susantad and Smith, 2008). Hence, we intended to target LamR’s action solely as the laminin receptor, so we utilized anti-LamR IgG to interrupt LamR activity. We used commercially available antibodies against sequences between 254 and 290 amino acids of LamR (LamR_254-290_), because the usage of IgG1-iS18, which target part of the immunogen sequence within this region (Supplementary Figure S2), strongly inhibits the LamR action as laminin-1-receptor (Omar et al., 2012; Rebelo et al., 2016). Before performing the functional tests, we verified the specificity of anti-LamR_254-290_ IgG using two methods–ICC and Western blot. Analysis of transfected chicken embryonic fibroblasts (DF-1) with a plasmid coding for LamR indicated a higher level of LamR in these cells in comparison to CTRL cells - transfected with the control plasmid (Supplementary Figure S3A–B). This showed that anti-LamR_254-290_ IgG is specific towards native LamR. Western blot analysis performed on DRG lysates revealed only one ca. 40 kDa band (expected for LamR) and no unspecific bands (Supplementary Figure S3C). This proved the specificity of anti-LamR_254-290_ IgG towards denatured protein as well. By using Western blot analysis, we showed that anti-LamR IgG did not change the expression of *RPSA* on protein level in DRG grown with the addition of IKVAV or laminin-1 (Supplementary Figure S3D).

**FIGURE 3:**
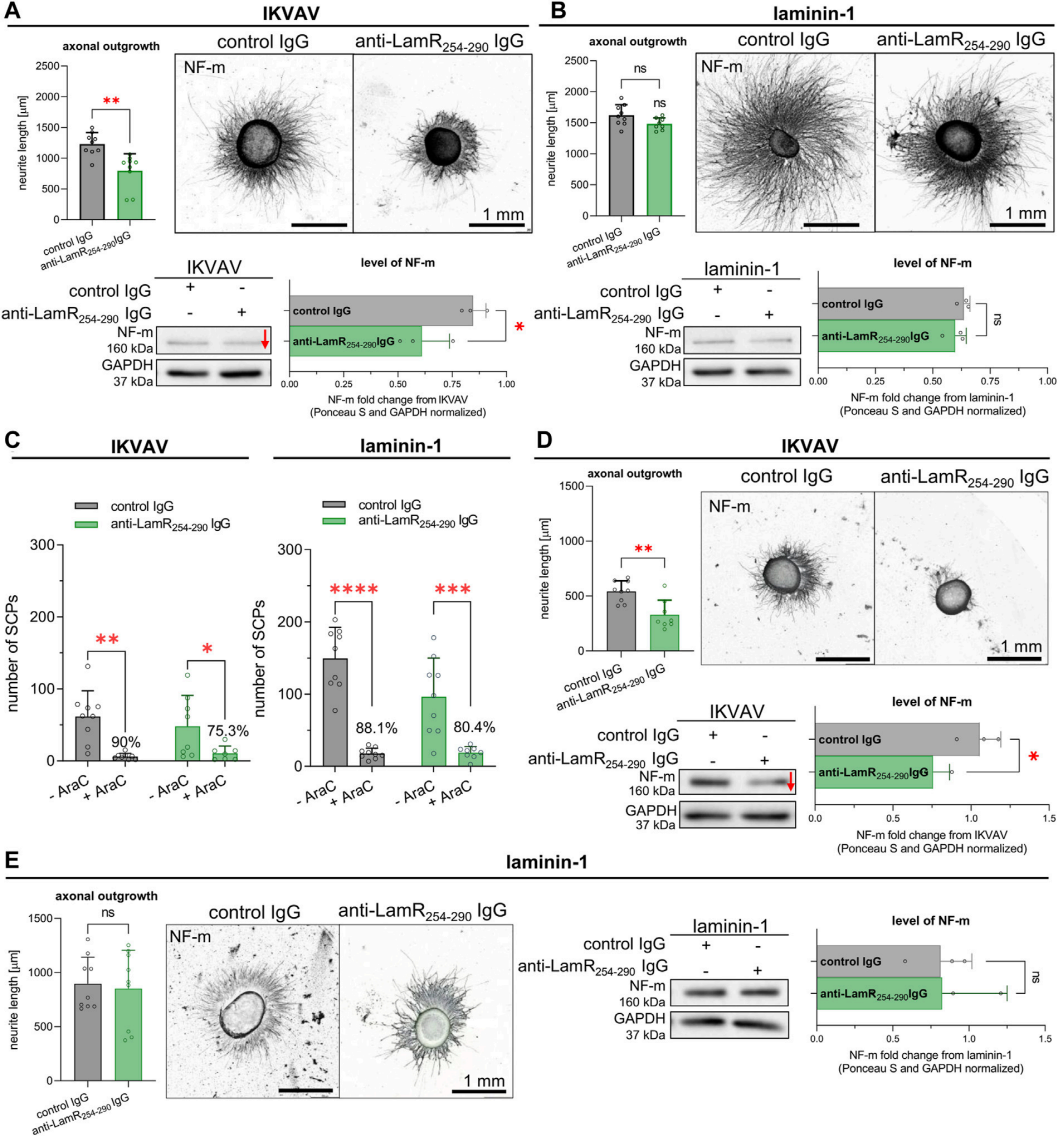
The impact of LamR on IKVAV- or laminin-1-dependent DRG neurite outgrowth in the presence and absence of SCPs. DRGs were cultured on a PDL-covered surface with IKVAV or laminin-1 **(B)** for 48 h. Antibodies directed against LamR were used to interrupt LamR activity, while control IgG were taken for control conditions. **(A, B)** Microphotographs of representative DRG growing with **(A)** IKVAV or **(B)** laminin-1, with quantitative analyses showing axonal outgrowth and the level of NF-m (*n* = 9); unpaired Student’s t-test. **(C)** The quantitative examination of SCPs number in DRG culture growing with or without Ara-C depicting the effect of LamR inhibition on axonal outgrowth. Data are shown as mean ± SD; Two-way ANOVA with Šídák’s test; (*n* = 9); **(D, E)** Representative DRG with quantitative analysis showing axonal outgrowth and the level of NF-m, both upon eliminating SCPs from culture by applying Ara-C, adequately to **(A, B)**. In axonal outgrowth assay, points represent the average length of ten of the longest neurites per DRG. For Western blot analysis, the points are the values for separate sets of samples. *p* ≥ 0.05 (ns); *p* < 0.05 (*), *p* < 0.01 (**); *p* < 0.001 (***); *p* < 0.0001 (****); The images were converted to black and white. For densitometry analysis, data were normalized to the Ponceau S and anti-GAPDH staining of membranes. Whole membranes stained with Ponceau S and anti-GAPDH IgG are shown in Supplementary Figure S6.

Finally, we verified the impact of LamR action on DRG axonal outgrowth in terms of laminin-1 or IKVAV stimulation using specific anti-LamR_254-290_ IgG. As a control condition, we used control antibodies from non-immunized mice–normal mouse IgG. Surprisingly, axonal outgrowth assay together with Western blot analyses revealed that DRG supplemented with IKVAV and anti-LamR_254-290_ IgG had significantly shorter axons and decreased level of NF-M (the level of NF-m reflects the axonal extension) compared to the control DRG (Figure 3A). In contrast, DRG exposed to laminin-1 showed no difference between neurite length and the level of NF-m upon anti-LamR_254-290_ IgG administration vs. control IgG (Figure 3B). Our previous studies showed that the effect of laminin-1 on axonal elongation is independent of the laminin-1 administration way (Mrówczyńska and Mazur, 2021). Hence, we chose to stimulate the axonal extension with laminin-1 added to the medium in this study. We believe this approach is more consistent with *in vivo* conditions of PNS development when laminins exist in extracellular space, e.g., as a source of glial cell secretome (Chiu et al., 1991). This also provides more comparable conditions for using IKVAV which is not immobilized as well.

To investigate whether the diminished axonal outgrowth after LamR inhibition may be associated with an indirect impact on neurons through SCPs, DRG were cultured with the addition of laminin-1 or IKVAV, along with exposure to either anti-LamR_254-290_ or control IgG. Additionally, Ara-C has been applied to substantially decrease the proliferation, thus the number of SCPs in culture. Firstly, we calculated the number of SCPs present along axons based on SOX10 signal to confirm the presence of SCP after Ara-C treatment of DRG. The data indicated that 75%–90% of SCPs were eliminated from DRG culture (Figure 3C). Axonal outgrowth assay and Western blot analyses showed that despite the reduced number of SCPs, axonal outgrowth and the level of NF-m were still negatively affected upon perturbation of LamR activity in DRG growing in the presence of IKVAV (Figure 3D), but not laminin-1 (Figure 3E).

All of these demonstrated that dysfunction of LamR negatively affects the axonal extension of sensory neurons when DRG are stimulated with IKVAV. This impact was observed regardless of whether precursors of peripheral glial SCPs were present.

### 3.3 The sequence-specific role of LamR on IKVAV-dependent DRG axonal outgrowth

To verify whether the effect of modulated LamR activity on IKVAV-dependent neurite outgrowth is exclusive to the 254–290 aa region, we assessed additional experiments using anti-LamR IgG with immunogen overlapping with presumable another laminin-1 binding region (LamR_181-230_ IgG) (Supplementary Figure S2). Anti-LamR_181-230_ IgG exhibited specificity in Western blot analysis (Supplementary Figure S4A). ICC analysis using anti-LamR_181-230_ IgG revealed a different distribution of LamR in DRG compared to LamR_254-290_ IgG (Supplementary Figure S4B, C). Although both types of antibodies detect LamR in somas of sensory neurons, anti-LamR_181-230_ IgG revealed the presence of LamR in SCPs nuclei and neurites (Supplementary Figure S4B). Similarly to the anti-LamR_254-290_ IgG, staining with anti-LamR_181-230_ IgG demonstrated no LamR on the cell membrane (Supplementary Figure S4C). The differences in detected LamR’s localization may result from the varying exposition of these epitopes in different locations. Axonal outgrowth assay indicated no changes in axon length upon adding anti-LamR_181-230_ IgG (Supplementary Figure S5). Thus, we decided not to use these antibodies in further experiments because of no effect on axonal outgrowth.

We can also conclude here that the effect of LamR on IKVAV-dependent axonal outgrowth may be region-dependent and specific for 254–290 aa region of LamR in tested conditions.

### 3.4 Internalization of anti-LamR_254-290_ IgG

**FIGURE 4:**
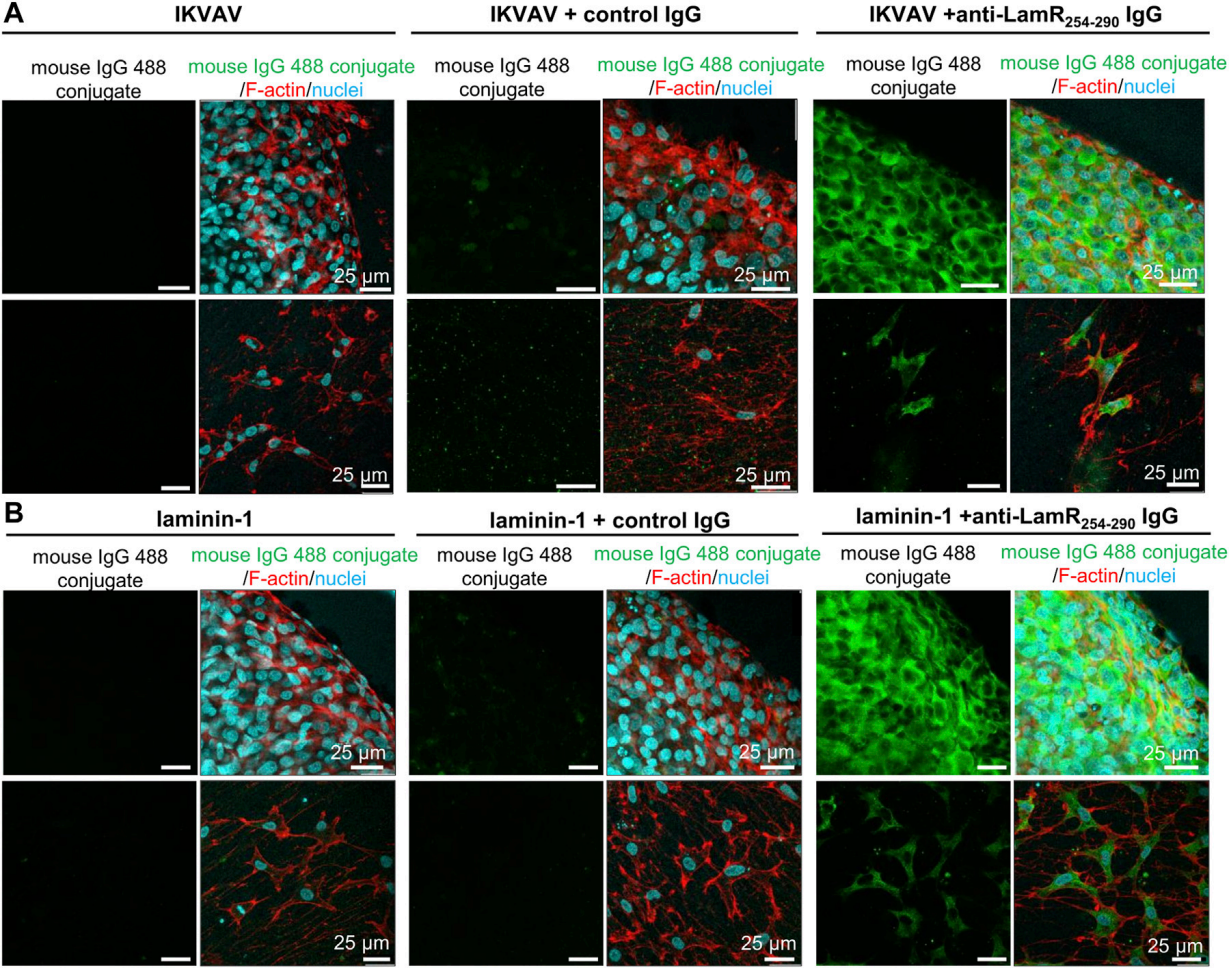
Uptake of anti-LamR_254-290_ IgG by neuronal cells and SCPs. DRG were fed with IKVAV **(A)** or laminin-1 **(B)** alone and additionally incubated with control or anti-LamR_254-290_ IgG. After 2 days, DRG were fixed and immunostained under cell membrane permeabilizing conditions using only secondary anti-mouse IgG-Alexa Fluor™ 488. DRG were stained with Hoechst 33342 and fluorescently labeled phalloidin to recognize DRG components. Images were taken within the same settings during one microscopic session. This experiment was repeated twice.

Due to the lack of LamR on the cell’s surface, we studied how anti-LamR_254-290_ IgG (targeting the LamR’s C-terminus) could affect intracellular LamR. Consequently, we investigated whether anti-LamR_254-290_ IgG could be internalized into DRG cells. For that, cultivation of DRG was conducted on PDL-coated glass in the presence of IKVAV or laminin-1, and additionally anti-LamR_254-290_ IgG or control IgG. DRG were then immunoassayed in permeabilized condition with secondary anti-mouse antibodies but with omitted incubation with primary antibodies. We observed no signal and a slight, non-specific signal (in somas) for anti-mouse IgG 488 conjugate in DRG cultured without any antibodies and with control IgG, respectively (Figures 4A, B). Interestingly, when DRG were supplemented with anti-LamR_254-290_ IgG, we observed a positive, analogous to LamR distribution (presented in Figure 1), signal for anti-mouse IgG 488 conjugate (Figure 4).

This shows that neurons, but also SCPs, internalize anti-LamR254-290 IgG, which then binds to the intracellular LamR (Figures 4A, B).

### 3.5 The impact of perturbing the LamR action on the level of laminin in the conditioned medium

**FIGURE 5:**
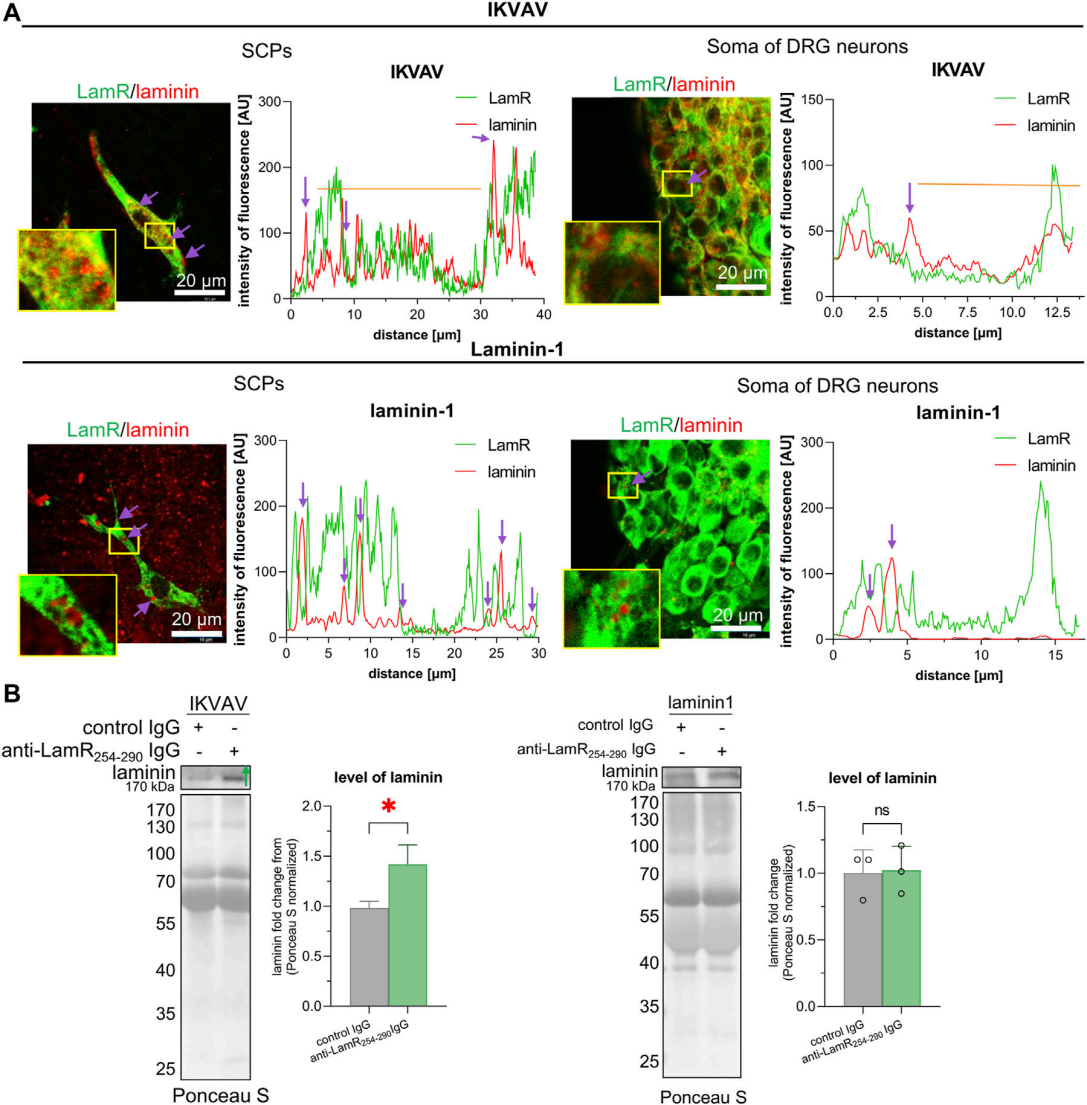
Evaluation of mutual localization of LamR and laminin-1 in DRG cells and the effect of LamR blocking on the level of extracellular laminin. **(A)** Representative images of double-labeled LamR and laminin-1 DRG, with the plots displaying the fluorescence distribution showing their colocalization. DRG were cultured with IKVAV or laminin-1 for 2 days before immunostaining. Purple arrows indicate the areas with no LamR and laminin-1 colocalization, whereas orange lines show positive colocalization in marked cell areas. Pictures were captured using identical settings during one microscopic session. **(B)** The laminin secretion by DRG cells, which were cultured with control or anti-LamR. DRG were cultured on coverslips coated with PDL with IKVAV or laminin-1 for 48 h. Then, conditioned media were collected and analyzed using the Western blot technique to detect laminin-1. *p* < 0.05 (*); unpaired Student’s *t*-test; (*n* = 3). The points are the values for separate sets of samples harvested from separate experiments. For densitometry analysis, data were normalized to the Ponceau S-stained membranes.

Because LamR binds laminin and we did not note the presence of LamR on the plasma membrane, we verified whether the intracellular localization of LamR and laminin was similar. Double staining for LamR and laminin showed that LamR exposed strong intracellular colocalization with laminin-1 both in SCPs and somas of DRG neurons when supplemented with IKVAV (Figure 5A). Nonetheless, some laminin-rich puncta lacked such colocalization. DRG growing with the addition of laminin-1, on the other hand, revealed minimal LamR-laminin-1 colocalization in neurons and glial precursors (Figure 5A). Additionally, using Western blotting analysis, we indicated that DRG cells growing with the addition of IKVAV produce and secrete laminin to the medium (Figure 5B). Moreover, the level of secreted laminin increased in the conditioned medium upon perturbation of LamR activity in the presence of IKVAV (Figure 5B). However, modulation of LamR activity did not affect the level of laminin in the medium rich in this ECM protein (Figure 5B).

These experiments showed that LamR apparently colocalizes intracellularly with laminin, which is supposedly secreted, but apparently not with internalized laminin. On the contrary, when there is a lot of laminin in the environment, the DRG cells do not have to produce laminin on their own. That is why we do not observe intracellular colocalization between LamR and laminin. Furthermore, diminished axonal elongation after affecting LamR activity is accompanied by changes in laminin secretion or internalization by DRG cells in the presence of IKVAV.

### 3.6 The influence of LamR activity impairment on DRG cell adhesion and cellular signaling associated with axonal outgrowth

**FIGURE 6:**
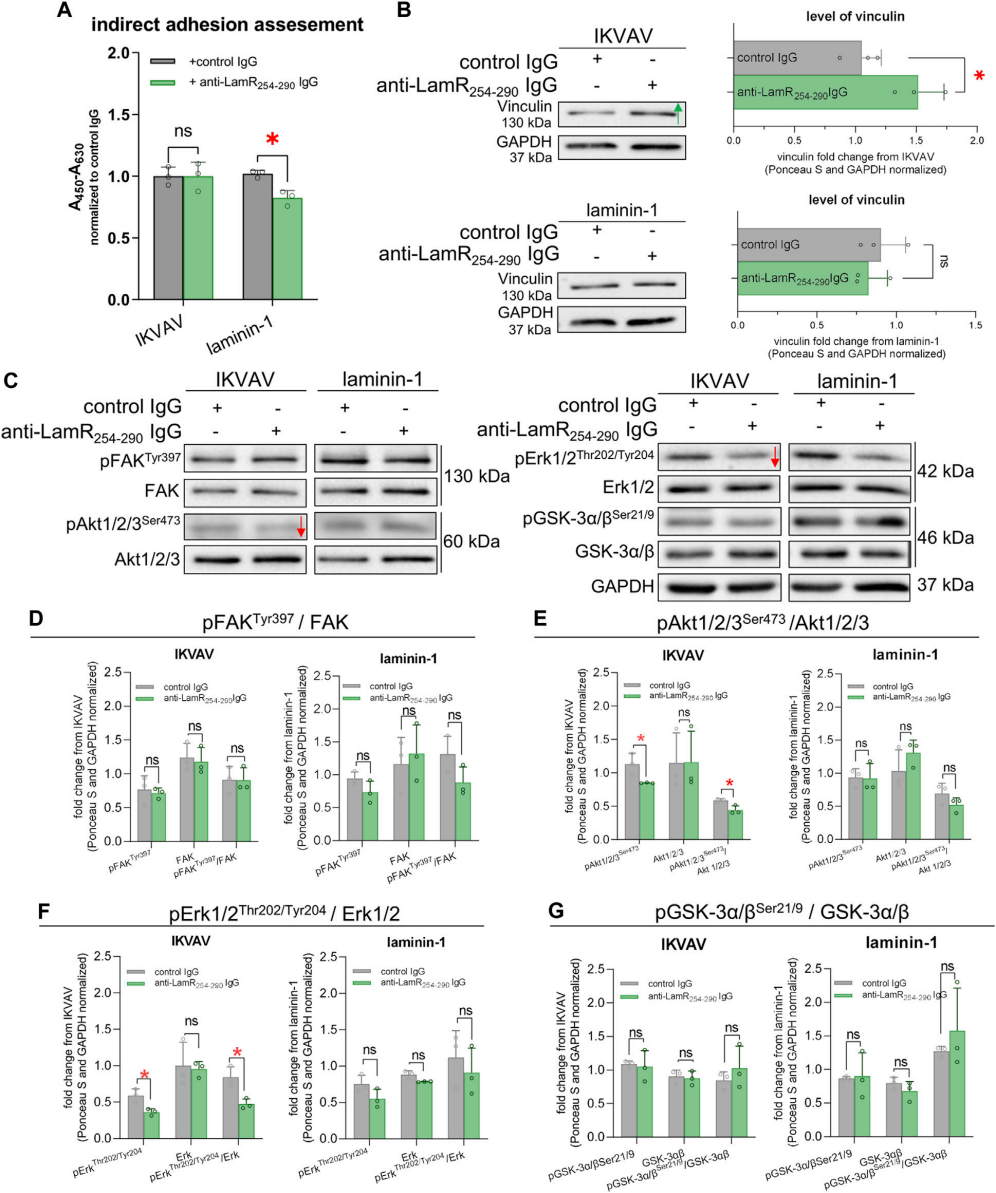
The consequence of LamR-blocking on DRG axonal adhesion and signaling pathways associated with axonal growth. DRG were cultivated with IKVAV or laminin-1 and with anti-LamR_254-290_ or control IgG for 48 h and then analyzed. **(A)** The effect of anti-LamR_254-290_ IgG on the adhesive properties of DRG cells. Measurement was done with a modified XTT assay. Graphs show mean ± SD. Each point represents normalized averaged data from 3 technical repeats taken from independent tests. *p* ≥ 0.05 (ns); *p* < 0.05 (*); Two-way ANOVA with Šídák’s test; (*n* = 3). **(B)** Representative immunoblots and quantification of the level of vinculin. Data represent fold change mean ± SD from three independent sets of samples. *p* ≥ 0.05 (ns); *p* < 0.05 (*); unpaired *t*-test; (*n* = 3). **(C)** Representative immunoblots showing the level of GSK-3α/β, FAK, Akt1/2/3, and Erk1/2 and their phosphorylated versions. **(D–G)** Quantitative analyses of immunoblots showing the total level and phosphorylation level of **(D)** FAK, **(E)** Akt1/2/3, **(F)** Erk1/2, and **(G)** GSK-3α/β. Data represent fold change mean ± SD from three independent sets of samples. *p* ≥ 0.05 (ns); *p* < 0.05 (*); unpaired *t*-test; (*n* = 3). Red arrows generally represent decreased protein levels. The images were converted to black and white. For densitometry analysis, data were normalized to the Ponceau S and anti-GAPDH staining of membranes. Corresponding control membranes stained with Ponceau S and anti-GAPDH IgG are shown in Supplementary Figure S6.

Adhesion is important for extension of axons during embryonic development. We assessed what is the effect of anti-LamR_254-290_ IgG on DRG cells on their adhesive abilities. We used a modified XTT assay that allowed to detect adhered DRG cells. Determination of cell adhesion is a common approach for studying LamR activity (Rebelo et al., 2016; Vilas-Boas et al., 2016). We found out that the incubation of laminin-supplemented DRG cells with anti-LamR_254-290_ IgG resulted in decreased adhesion when compared to the control (Figure 6A). The adhesion of DRG cells seeded in the presence of IKVAV and anti-LamR_254-290_ IgG was not altered (Figure 6A). Additionally, we analyzed the DRG lysates for the level of vinculin, which is involved in the adhesion process through the development and activity of adhesion-related structures named focal adhesions (FAs) (Humphries et al., 2007). We observed the increased level of vinculin upon the perturbation in LamR activity when DRG were cultured with IKVAV (Figure 6B).

Next, we deciphered the mechanisms responsible for decreased neurite outgrowth upon LamR targeting. We studied the level and the activity of proteins associated with laminin activity and signaling pathways involved in axonal outgrowth. Focal adhesion kinase (FAK), an element of the signaling pathways triggered by integrins and crucial for cell adhesion and migration (Mitra et al., 2005), was one of the proteins under evaluation. We also evaluated the activity Akt (PKB) - important for proliferation, survival, and growth processes; glycogen synthase kinase (GSK3α/β) - crucial for cell migration, adhesion, and division; and extracellular signal-regulated kinases (Erk1/2) - playing a vital role in axonal outgrowth and repair (Perron and Bixby, 1999; Kobayashi et al., 2006; De Toni-Costes et al., 2010; Tsuda et al., 2011). Using Western blot, we noticed a decreased level of phosphorylation of Akt1/2/3 and Erk1/2 upon anti-LamR_254-290_ IgG treatment of DRG treated with IKVAV compared to the control IgG (Figures 6C, E, F). The phosphorylation state of FAK and GSK-3α/β was not altered (Figures 6C, D, G). When the activity of LamR was manipulated by IgG in laminin-1 treated culture, there were no changes in the phosphorylation level of any of the tested proteins (Figures 6C–G). Corresponding loading controls are presented in Supplementary Figure S6.

Overall, the modulation of LamR action led to changes in adhesive properties in DRG cells and the level of vinculin as a protein involved in adhesion, depending on the type of ECM stimulator. Additionally, the action of LamR in IKVAV-dependent stimulation of neurites to elongation seemed to be associated with Akt1/2/3 and Erk1/2 signaling pathways.

### 3.7 LamR’s role in β1 integrin-dependent axonal outgrowth

Studies have shown that LamR may be a “co-receptor” for other membrane receptors. LamR and integrins may collaborate in the modulation of invasion and metastasis (Ardini et al., 1997; Wu et al., 2016). We were interested in finding out if the reported negative influence of LamR inhibition on DRG axon extension in the presence of IKVAV was attributed to an interruption in LamR-integrin or IKVAV-β1 integrin interactions. For this purpose, we combined anti-LamR_254-290_ IgG with the antibodies blocking (CSAT) or activating (TASC/9D11) β1 integrin. First, we confirmed the action of blocking and activating anti-β1 integrin antibodies (Supplementary Figure S7). CSAT IgG resulted in decreased axonal length in laminin-1 and IKVAV-treated DRG. However, TASC/9D11 IgG did not change the length of axons in the IKVAV-treated culture, but they resulted in increased axonal elongation in laminin-1-treated DRG (Supplementary Figure S7).

**FIGURE 7:**
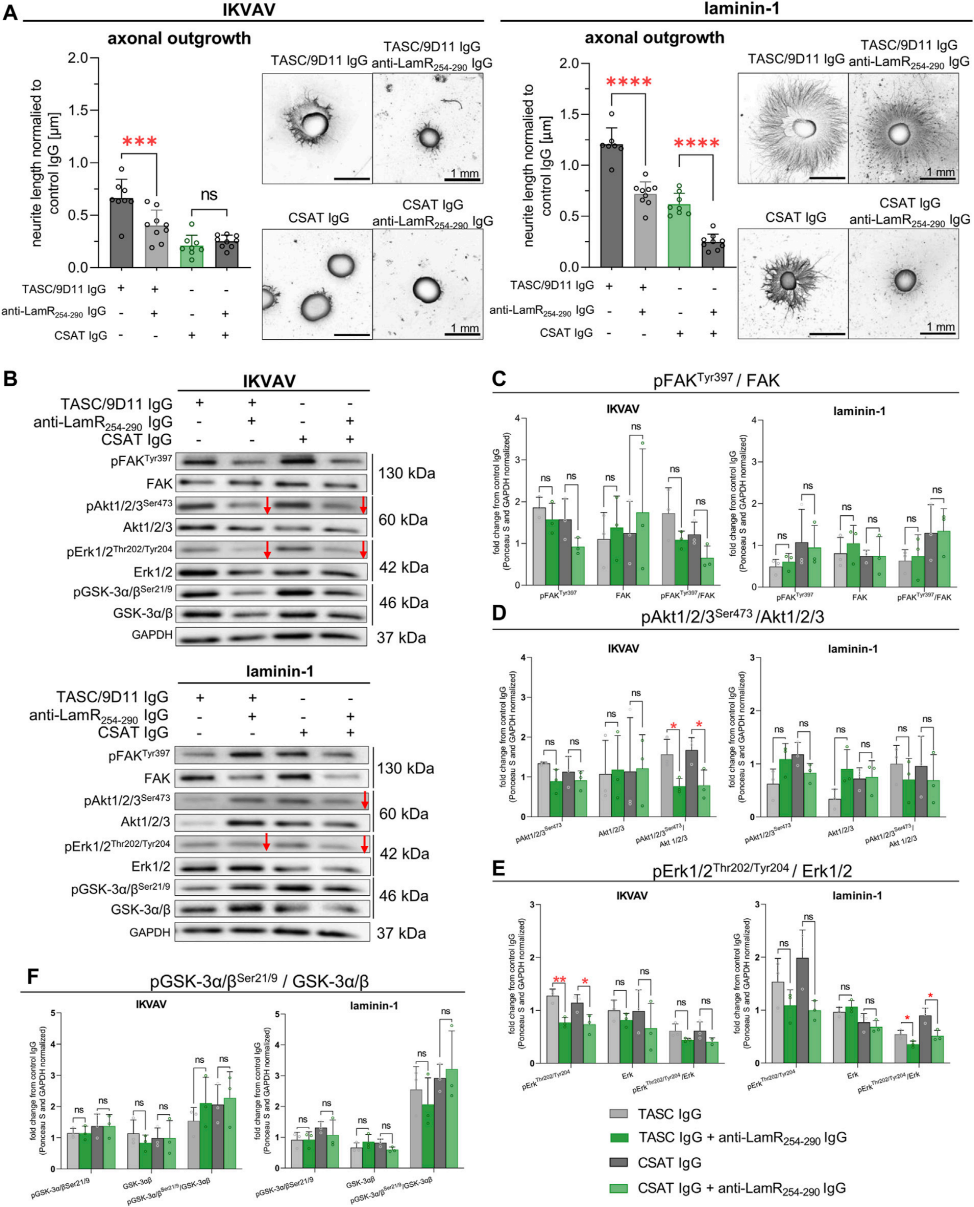
The impact of LamR on DRG neurite outgrowth in the context of β1 integrin activity. To activate β1 integrin, TASC/9D11 antibodies were used, while CSAT IgG were used to inhibit β1 integrin activity. β1 integrin was either inhibited or activated simultaneously with anti-LamR_254-290_ IgG treatment. **(A)** Axonal outgrowth analysis with representative photos of DRG. The data points represent the average length of the ten most extended neurites per DRG normalized to the averaged value for the control condition. Data are shown as mean ± SD; *p* ≥ 0.05 (ns); *p* < 0.001 (***); *p* < 0.0001 (****); one-way ANOVA with Šídák’s test (*n* = 9). **(B)** Representative immunoblots presenting the level and phosphorylation level of tested proteins. Red arrows represent decreased protein levels. **(C–F)** Quantitative analyses showing the total level and phosphorylation level of **(C)** FAK, **(D)** Akt1/2/3, **(E)** Erk1/2, and **(F)** GSK-3α/β. Data represent fold change mean ± SD from three independent sets of samples. *p* ≥ 0.05 (ns); *p* < 0.05 (*); *p* < 0.01 (**); one-way ANOVA with Šídák’s test; (*n* = 3). The images were converted to black and white. Data were normalized to the Ponceau S and anti-GAPDH stained membranes. Control membranes stained with Ponceau S and GAPDH are shown in Supplementary Figure S8.

Further on, we showed that additional treatment of laminin-stimulated DRG with anti-LamR_254-290_ IgG effectively decreased the axonal length independent of the way of modulation of activity of β1 integrin (Figure 7A, right panel). A combination of anti-LamR and TASC/9D11 IgG led to a decreased axonal outgrowth when DRG were exposed to IKVAV (Figure 7A, left panel). In the case of treatment of DRG with anti-LamR and CSAT IgG, we did not see any differences compared to the application of only CSAT IgG (Figure 7A, left panel), probably because CSAT on its own drastically reduced the axonal outgrowth so much that the DRG with shorter axons during ICC procedure detached due to insufficient adhesion surface to the substrate.

Subsequently, the signaling pathways responding to LamR dysfunction under β1 integrin-modulating conditions were studied. Figure 7B displays exemplary immunoblots, and Supplementary Figure S8 - loading controls. Analyses demonstrated that additional blocking of LamR in the presence of IKVAV caused a decrease in the relative level of pErk1/2 (a tendency) and pAkt1/2/3 compared to the modulation of β1 integrin activity on its own (Figures 7B, D, E). Within the presence of laminin-1, the decrease in the phosphorylation of Erk1/2 and Akt1/2/3 (a tendency) upon LamR blocking was observed when β1 integrin activity was dysregulated (Figures 7B, D, E), which again correlated with the effect of LamR activity on axonal outgrowth (Figure 7A). The level of pFAK^Tyr397^ and pGSK-3α/β^Ser21/9^ was not altered in all tested conditions (Figures 7B, C, F).

Overall, we show that LamR activity is vital for the outgrowth of DRG axons when β1 integrin activity is perturbed. However, when β1 integrin properly functions, LamR plays a minor role in regulating axonal outgrowth. Nevertheless, we observed that LamR regulates the elongation of axons by influencing Erk and Akt signaling in the presence of both IKVAV and laminin-1.

## 4 Discussion

The known role of LamR in the cell-laminin interaction would imply the presence of LamR on the cell membrane or even its secretion to the medium, as was shown previously (Romanov et al., 1994; Karpatová et al., 1996). Studies on human melanoma cells confirmed LamR’s exposure on the cell’s surface following laminin-1 stimulation (Romanov et al., 1994). The membranous presence of LamR in response to the bacterial toxin LPS lipopolysaccharide was shown on murine macrophages as well. Oppositely, the polyphenol epigallocatechin gallate from green tea prompted LamR internalization into the cell (Ren et al., 2014). Studies on PC12 cells also indicated LamR internalization via endosomal vesicles in response to laminin-1 or YIGSR (Davis et al., 2017). All these studies proved how dynamic LamR shuttling is between the outer side of the plasma membrane and the interior of a cell. Here, we demonstrated exclusive intracellular LamR localization in DRG sensory neurons and SCPs in tested conditions. We would like to highlight the importance of using unified protocols for sensitive procedures, such as immunocytochemical procedures in non-permeabilizing conditions. Diversity in performing the protocols may lead to false results. An example is the commonly used fixation of cells with formaldehyde, which is known to cause the formation of pores in the cell membrane and false positive signals from detected proteins believed to be present on the cell’s surface. We propose that the internalization of laminin-1 or its intracellular presence due to production by DRG cells could explain the observed intracellular colocalization between LamR and laminin-1. Alternatively, LamR:laminin-1 complexes could be internalized so quickly that it did not allow for the detection of LamR on the cell surface. However, we did not detect colocalization between LamR and laminin-1 in DRG cells growing in the surplus of laminin-1. Thus, it is rather an improbable situation. Finally, the mechanisms of intracellular LamR action toward axonal outgrowth presented in this paper may be related to the ribosomal or cytoskeletal-related activity of LamR, but this requires more studies. Summarizing, others proved that LamR can interact with laminin-1 and modulate its binding to the cells when present on the cell membrane, being secreted or added, e.g., as soluble peptide (peptide G) to the culture medium (Malinoff and Wicha, 1983; Karpatová et al., 1996; Magnifico et al., 1996; Berno et al., 2005). Here, we show that the intracellular LamR’s role in developing DRG apparently depends on the interaction with intracellular laminin-1 when laminin-1 is not externally provided.

Currently, there is no specific inhibitor for LamR protein on the market. Several lines of evidence indicate that NSC47924 {1-[(4-methoxyanilino)methyl]-2-naphthol} influences the laminin-1:LamR interactions in HEK296 cells. This drug suppresses cell adhesion, migration, and invasion on laminin-1 (Pesapane et al., 2015). Others showed that NSC47924 impacts the cell surface localization of LamR and its interplay with cellular prion protein, which could be crucial for targeting various neurodegenerative diseases (Sarnataro et al., 2016). Unfortunately, NSC47924 seems to be an unspecific inhibitor of LamR since it also acts on phosphatases from the PHLPP family (Sierecki et al., 2010). Consequently, there is an increasing interest in the LamR blocking strategy, which uses antibodies targeting LamR. Therefore, we followed this trend and used antibodies targeting the 254–290 amino acids sequence (LamR_254-290_) to interrupt the biological activity of LamR connected with laminin-1 interaction. We intentionally decided not to downregulate the *RPSA* expression to not disturb ribosome functioning, as LamR (*RPSA*) is an essential ribosomal protein (McCaffery et al., 1990). As mentioned previously, IgG1-iS18 antibodies targeting a similar fragment (272–280 aa) inhibit the laminin-LamR interaction. IgG1-iS18 binding to LamR within the 272–280 aa sequence is hypothesized to indirectly alter LamR’s laminin-binding fragment’ affinity for the ligand by modifying their conformation (Rebelo et al., 2016). We speculate that anti-LamR_254-290_ IgG could function in the same way. Interestingly, we proved that anti-LamR_254-290_ IgG is internalized into DRG neurons and peripheral glial cells, potentially interfering with intracellular LamR. The primary obstacle to the therapeutic application of intracellular antigen-targeting antibodies is its difficulty in penetrating cells or reaching the cytoplasm from endosomes. Nevertheless, it has been proved that anti-DNA antibodies (3E10 and 3D8) can internalize into cells, escape from the caveosome, and locate within the cytosol or nucleus, respectively (Jang et al., 2009; Rattray et al., 2018). Apparently, anti-LamR_254-290_ IgG may reach intracellular LamR, affecting its activity similarly. However, the specific mechanism of such action remains unclear and requires further research. To summarize, since IgG1-iS18 antibodies represent a promising tool for treating prion diseases, Alzheimer’s disease, or different types of cancers (Chetty et al., 2014; Rebelo et al., 2016), this indicates that the anti-LamR antibodies we used could have therapeutic potential in diseases associated with abnormalities in the PNS development and functioning.

For the first time, we show the important role of LamR in IKVAV-stimulated DRG axonal outgrowth. The IgG specific for LamR_254-290_ caused diminished growth of axons in this condition. Additionally, LamR_254-290_ negatively affected the level of NF-m, probably as an effect of shorter neurites. NF-m also connects with other cytoskeletal components along neurites, providing stability and transport in extending axons (Frappier et al., 1992; Yuan et al., 2017). It leads to the conclusion that LamR could contribute to both axonal expansion and neuronal cytoskeletal stability. Nevertheless, anti-LamR_254-290_ IgG did not disrupt axonal outgrowth and change the level of NF-m when DRG were growing in the surplus of laminin-1. Others have already shown that various glial cells, such as astrocytes, oligodendrocytes, and Schwann cells, can influence the surrounding microenvironment by secreting neurotrophic factors or ECM proteins to modulate axonal growth (Liesi and Silver, 1988; Jacobs, 2000; Thompson and Buettner, 2006; Liu et al., 2015). We investigated whether affected axonal outgrowth due to LamR’s dysfunction depends on SCPs. However, after removing SCPs from DRG cultures, similar to the conditions in which SCPs were present, LamR’s inhibition affected axonal outgrowth with IKVAV but not laminin-1. The persistence of diminished axonal outgrowth in the presence and absence of SCPs suggests that LamR directly affects sensory neurons, while SCPs are irrelevant to this process. Our data also shows that laminin can be produced by neuronal cells in the lack of externally delivered laminin.

Our study revealed that the stimulation of DRG with IKVAV differs compared to the native laminin, even if IKVAV is known as an active site of laminin. Remarkably, laminin distribution varied between DRG cultivated with IKVAV or laminin-1. In the presence of IKVAV, laminin-1 was produced by DRG cells and co-localized with LamR inside the sensory neurons and SCPs. Additionally, anti-LamR_254-290_ IgG application was associated with higher levels of laminin-1 in the conditioned medium. In the presence of external laminin-1, its intracellular localization was punctate and divergent to LamR, and the level of laminin in the medium was not changed. We think that these differences could explain the negative action of LamR_254-290_ IgG administration on the elongation of neurites of DRG growing in the presence of IKVAV, but not laminin-1. This proved also that LamR activity can also modulate the level of extracellular laminin. As it was mentioned, others proved LamR (cell membrane found and shed form of LamR) activity towards laminin remodeling (Karpatová et al., 1996; Berno et al., 2005), but here we showed the role of intracellular LamR in this process. However, in the case of laminin-treated DRG no effect of LamR blocking on axonal outgrowth could be explained by the presence of several laminin-1 binding regions in LamR that needed to be simultaneously blocked (Kazmin et al., 2000; Digiacomo and Meruelo, 2016). But the region 181–230 aa is not one of them as we showed that LamR_181-230_ IgG (carrying the immunogen sequence of overlaps with laminin-1 binding regions) could not affect either IKVAV- or laminin-1-dependent axonal outgrowth. The ideal would be to test antibodies targeted in other regions of LamR. However, commercial access to the highly specific, chicken protein-oriented antibodies is strongly limited, which makes it difficult to perform such experiments.

Laminins promote axon elongation largely via adhesion (Woo and Gomez, 2006). Here, we found out that anti-LamR_254-290_ IgG negatively impacted DRG cell adhesion in the presence of laminin-1. We propose that the disruption of LamR:laminin-1 interactivity may weaken the adhesion of DRG cells, thus facilitating their unchanged elongation. On the other hand, no changes in the adhesion of DRG grown with IKVAV, together with a lifted level of vinculin, indicated the involvement of LamR in ECM-cell interaction and adhesion. However, this process was independent of FAK activity. It has already been shown that LamR colocalizes with vinculin in neural crest cells (Fu et al., 2020), but we showed the impact of LamR action on producing this focal adhesion protein for the first time. Furthermore, we noticed the altered activity of Akt1/2/3 and Erk1/3, which are known to regulate axonal outgrowth (Tucker et al., 2008; Zhao et al., 2009; Tsuda et al., 2011; Diez et al., 2012; Jin et al., 2018). This, together with diminished axonal outgrowth in the IKVAV-dependent culture of DRG, suggests that the mechanism of LamR’s action is associated with regulating these intracellular pathways. The connection between LamR activity and MAPK/Erk, Akt, and GSK signaling pathways has already been determined. It was shown that the blockage of LamR increased the activity of p38 MAPK, Erk1/2, and PI3K/Akt signaling pathways in endothelial cells and astrocytes (Kim et al., 2020). Additionally, RPSA inhibition resulted in the inactivation of MAPK/Erk signaling in mouse hypothalamic neuronal cells, which linked it with autophagy (Bhattacharya et al., 2020). Here, we show that the effect of LamR dysregulation relates to changes in levels and activation of Akt1/2/3 and Erk1/2 signaling, which strongly contribute to the axonal elongation in the embryonic sensory neurons of DRG.

Our findings show the connections between LamR and β1 integrin activity in regulating axonal outgrowth in DRG. Inhibition of LamR’s action led to decreased neurite elongation and negative in response to IKVAV and laminin-1 when β1 integrin was either inhibited or activated. Consequently, together with the diminished length of axons, the decrease in the activity of Erk1/2 and Akt1/2/3 was altered upon application of anti-LamR_254-290_ IgG. This again emphasizes that LamR may be crucial for modulating axonal outgrowth through Erk1/2 and Akt1/2/3 signaling. Our data show that the activity of β1 integrin in unperturbed DRG growth is more important and masks the effect of LamR activity in this process. Finally, if β1 integrin activity is somehow affected, LamR starts to play a major role in the regulation of laminin-1-dependent axonal outgrowth.

**FIGURE 8:**
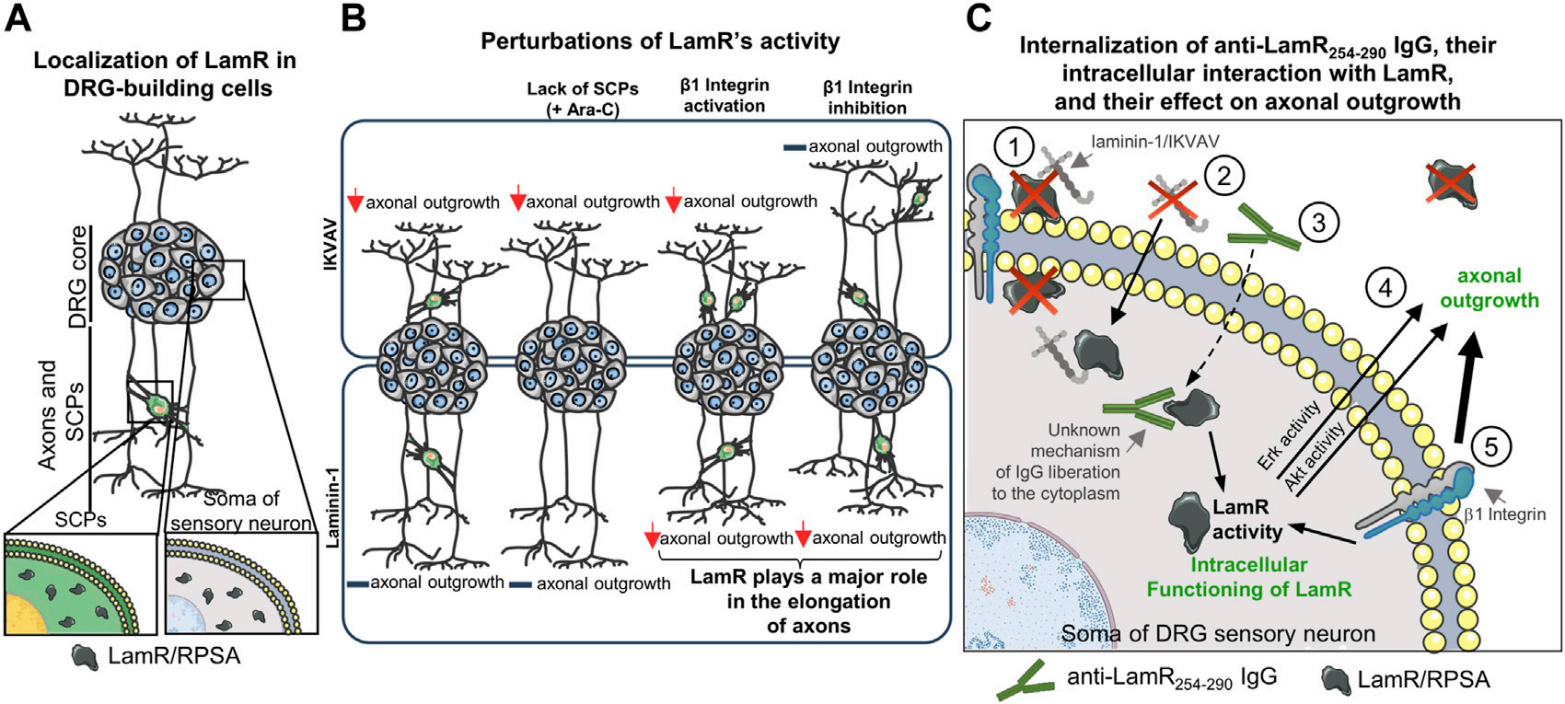
Scheme illustrating how affecting the LamR activity influences the DRG axonal outgrowth in the presence of laminin and laminin-mimicking peptide - IKVAV. **(A)** LamR, detected with anti-LamR_254-290_ IgG, is localized only intracellularly in DRG neurons and SCPs. **(B)** The effect of perturbation of LamR activity on axonal outgrowth in various conditions. Dysregulation of LamR activity negatively impacts axonal growth of sensory neurons upon stimulation of DRG with IKVAV, but not laminin-1, regardless of the presence of SCPs. When DRG were grown in the presence of laminin-1, the importance of LamR toward elongation of axons is minor until dysregulation of β1 integrin activity. **(C)** Proposed mechanism of the action of LamR and LamR_254-290_ IgG on axonal outgrowth. (1) Since LamR is absent on DRG cell membranes, it is rather improbable that LamR interacts with integrins or binds to laminin-1/IKVAV. (2) Therefore, LamR may act through its intracellular function by interaction with intracellular laminin, which is located intracellularly when there is no external source of laminin-1 in DRG culture. (3) Anti-LamR_254-290_ IgG internalize into sensory neuron somas and binds intracellular LamR. It remains to be solved what is the mechanism of that. (4) The role of LamR in DRG axonal outgrowth may relate to the impact on Erk1/2 and Akt1/2/3 activity, which are known to be involved in the regulation of axonal outgrowth. (5) β1 integrin-dependent signaling dominates LamR activity regarding axonal outgrowth stimulated by laminin-1.

Overall, we showed the complex function of intracellular LamR in regulating axonal outgrowth in chicken embryonic DRG (summarized in Figure 8). We claim that intracellular LamR is an important mediator in the crosstalk between laminin-1/IKVAV and β1 integrin, influencing the balance of signaling events necessary for optimal axonal outgrowth. Moreover, our data implies that the influence of LamR on the extension of neurites comes to light when the proper action of β1 integrin is affected. Finally, Erk1/2 and Akt1/2/3 signaling seems to be the key signaling pathways impacted by anti-LamR IgG administration. In our view, it would be interesting to further focus on the mechanisms of anti-LamR_254-290_ IgG entering the cell and binding to intracellular LamR. We think that our study is the groundwork for future research into the therapeutic potential of targeting LamR in the context of neuronal development and even regeneration. LamR-targeted inhibition of axonal outgrowth could help regulate/correct the abnormal or excessive growth of neurites during neurodevelopmental diseases such as neuropathies or pathological nerve regeneration - neuromas. The knowledge of which regions of LamR interact with laminin could lead to the design of drugs modulating the desired response of neuronal cells in the context of axonal elongation or activation of Akt or Erk kinases without affecting ribosome activity.

## Data Availability

The raw data supporting the conclusions of this article will be made available by the authors, without undue reservation.
