# Supplementary material for: Non-integrin laminin receptor (LamR) plays a role in axonal outgrowth from chicken DRG via modulating the Akt and Erk signaling
Source: Front Cell Dev Biol. 2024 Jul 31;12:1433947. doi: 10.3389/fcell.2024.1433947 (PMC11322362; doi:10.3389/fcell.2024.1433947)
Supplement: Supplementary file 1 [file DataSheet1.PDF]

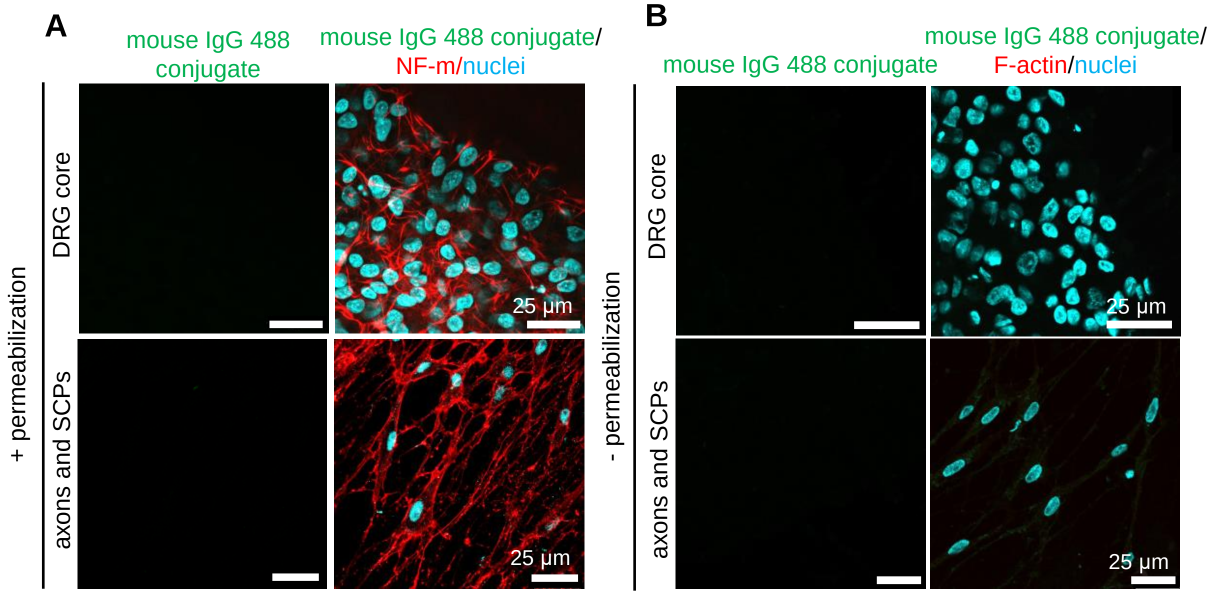

Supplementary Figure 1. Negative controls for immunocytochemical stainings which are shown in Figures 1 and 2A. DRG were cultured on coverslips coated with PDL and laminin-1 for two days and stained under permeabilizing (A) and non-permeabilizing (B) conditions. DRG were immunostained without utilizing primary antibodies against anti-LamR. Samples were incubated with goat anti-mouse-Alexa Fluor™ 488 antibodies, Hoechst 33342 dye, and fluorescently labeled phalloidin to detect cell nuclei and F-actin. The images depict somas and axons of sensory neurons and SCPs.

```
RPSA_CHICK      MSGGLDVLQMKEEDVLKFLAAGTHLGGTNLDFQMEQYIYKRKSDGIYIINLKRTWEKLLL   60
RPSA_HUMAN      MSGALDVLQMKEEDVLKFLAAGTHLGGTNLDFQMEQYIYKRKSDGIYIINLKRTWEKLLL   60
                ***.********************************************************

RPSA_CHICK      AARAIVAIENPADVSVISSRNTGQRAVLKFAAATGATPIAGRFTPGTFTNQIQAAFREPR   120
RPSA_HUMAN      AARAIVAIENPADVSVISSRNTGQRAVLKFAAATGATPIAGRFTPGTFTNQIQAAFREPR   120
                ************************************************************

RPSA_CHICK      LLVVTDPRADHQPLTEASYVNIPTIALCNTDSPLRYVDIAIPCNNKGAHSVGLMWWMLAR   180
RPSA_HUMAN      LLVVTDPRADHQPLTEASYVNLPTIALCNTDSPLRYVDIAIPCNNKGAHSVGLMWWMLAR   180
                *********************:**************************************

RPSA_CHICK      EVLRMRGTISREHPWEVMPDLYFYRDPEEIEKEEQAAAEKAVTKEEFQTEWTAPAPEFTA   240
RPSA_HUMAN      EVLRMRGTISREHPWEVMPDLYFYRDPEEIEKEEQAAAEKAVTKEEFQGEWTAPAPEFTA   240
                ************************************************ ***********

RPSA_CHICK      PPQPEVADWSEGVQVPSVPIQQFPTEDWSAQPATEDWSAAPTAQATEWVGTTTEWS       296
RPSA_HUMAN      -TQPEVADWSEGVQVPSVPIQQFPTEDWSAQPATEDWSAAPTAQATEWVGATTDWS       295
                --************************************************:**:**
```

IPC…LAR - peptide G (161-180 aa), laminin binding region

RDP…FQT - peptide 205 – 229 aa, laminin binding region

TEDWSAAP - peptide containing DWS repeat, potential laminin binding region, the target sequence for IgG1-iS18 antibody against LamR

QVP…WVG – target sequence for anti-$LamR_{254-290}$ IgG

EVL…QTE – target sequence for anti-$LamR_{218-230}$ IgG

Supplementary Figure 2. Alignment of amino acid sequences of chicken and human LamR. Red boxes represent the differences between chicken and human protein sequences. The laminin-binding sequences are shown as yellow and green areas. The yellow marked sequence contains peptide G (161-180 aa), whereas the green highlights 205-229 aa peptide. Peptide TEDWSAAP, which potentially binds laminin and is a target sequence for IgG1-iS18 antibody against LamR, is marked as a dark grey area. The target sequence for anty-$LamR_{254-290}$ IgG is represented in light grey. Sequences are based on primary accession number P50890 for chicken LamR and P08865 for the human version of LamR (The UniProt Consortium database)

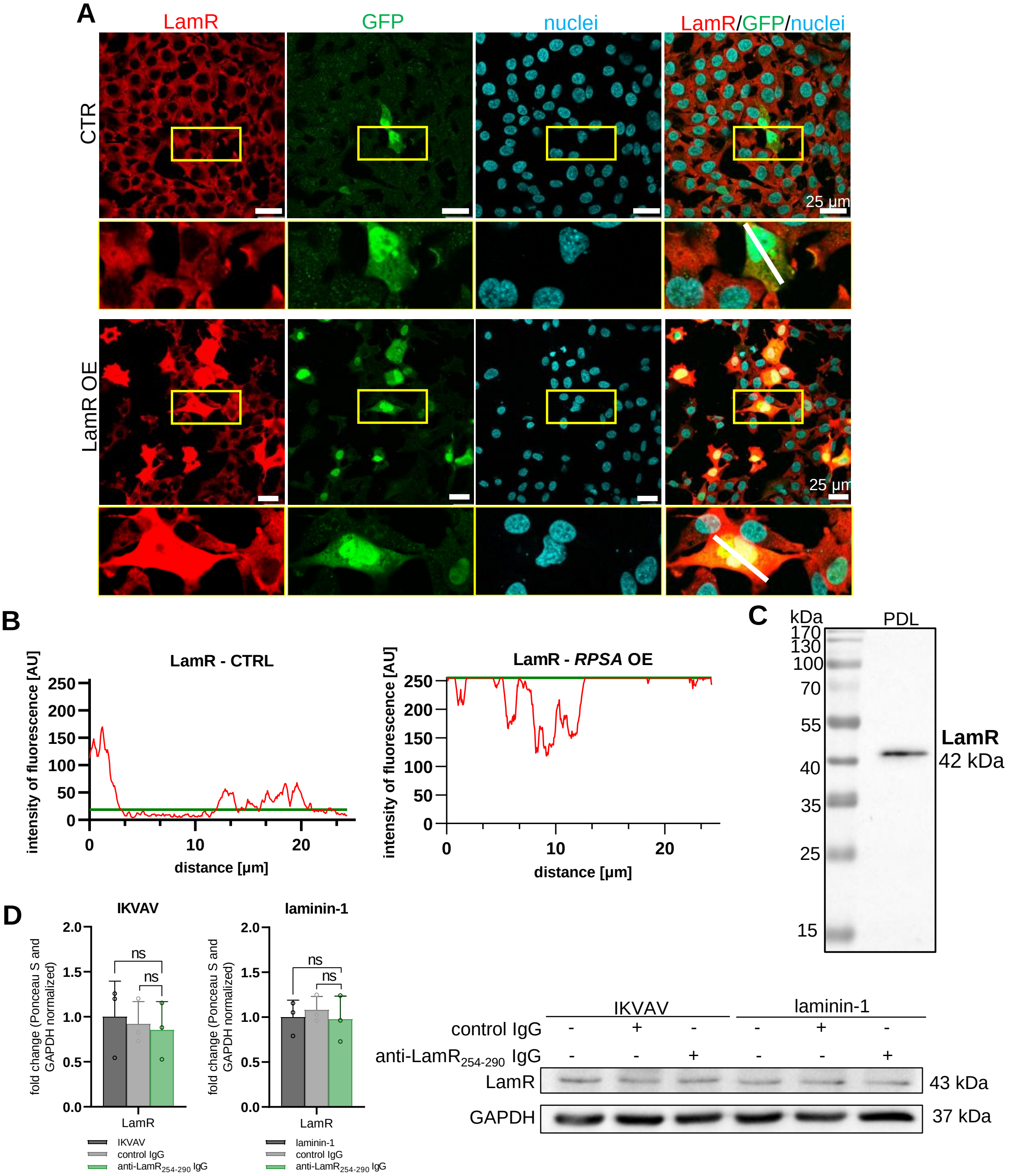

Supplementary Figure 3. Anti-LamR$_{254\text{-}290}$ antibodies specificity. **(A, B)** Immunocytochemical analysis of chicken embryonic fibroblasts DF-1 transfected with control plasmid or plasmid coding for LamR. Cells were immunostained using anti-LamR$_{254\text{-}290}$ antibodies to verify the specificity of these IgG, anti-GFP antibodies to detect transfected cells, and a DNA-specific dye – Hoechst 33342. Insets enlarge transfected cells, showing LamR increased signal in transfected with plasmid coding for LamR cells compared to the control cells. Histograms confirm the level of LamR intensity in transfected cells. A white line was drawn on an image of the representative cell, and the fluorescence histograms, which show the signal intensities, were plotted by applying this as a basis. **(B)** Histograms showing the intensity of fluorescence of LamR across cells (red). Green lines represent the median of the intensity of fluorescence calculated based on all intensity of fluorescence values across the cell. Pictures were taken with the same settings during the same microscopic session. White lines shows how histograms were performer across the cell. **(C)** Detection of LamR using anti-LamR$_{254\text{-}290}$ antibodies in DRG lysates growing in control conditions – on a PDL-coated surface for 48 h. **(D)** Evaluation of *RPSA* gene expression by estimation of LamR protein level in DRG cultured without any antibodies, with control IgG or with anti-LamR$_{254\text{-}290.}$ GAPDH and PonceauS acted as a control.

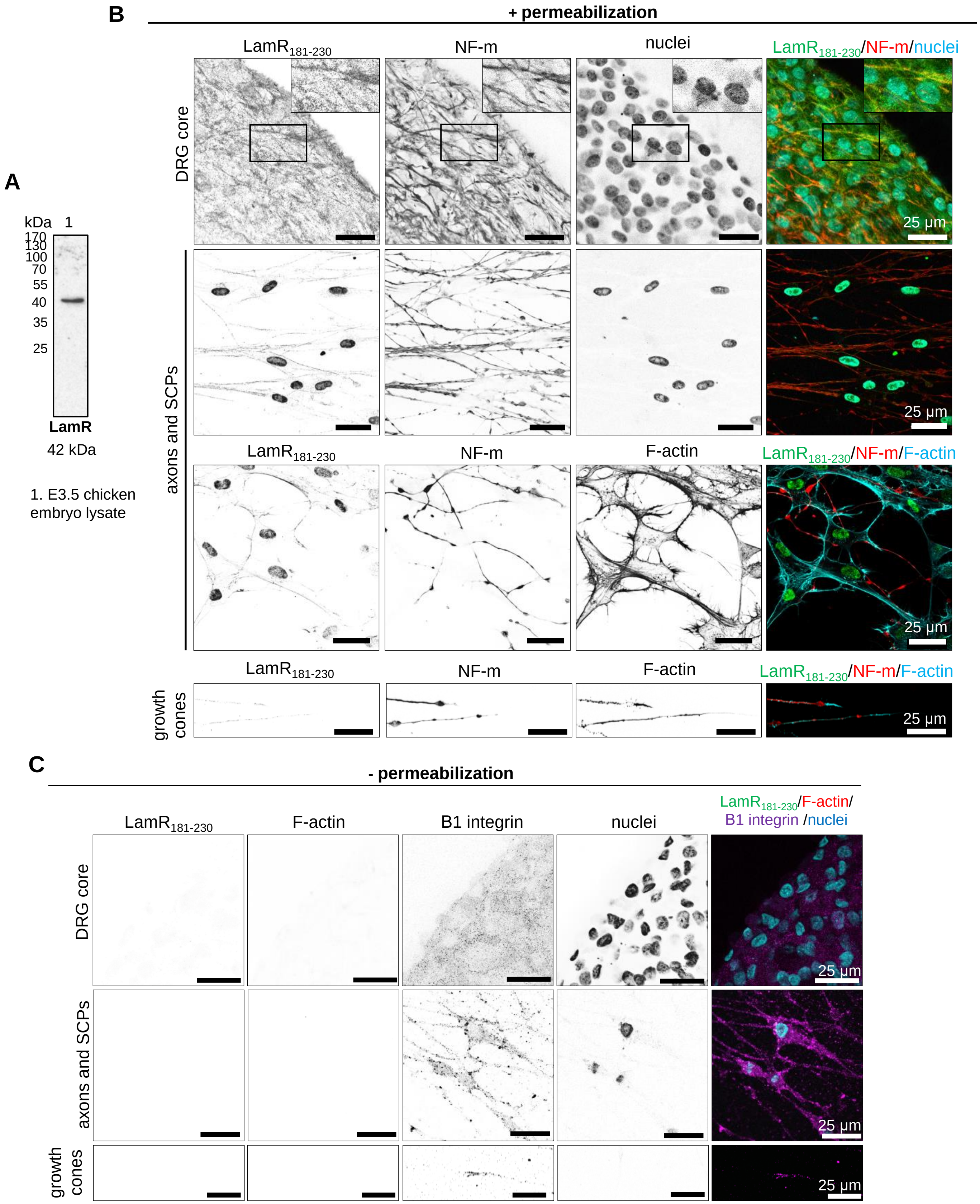

Supplementary Figure 4. Evaluation of LamR's localization in DRG cells and on the plasma membrane of these cells with the application of anti-LamR$_{181\text{-}230}$ IgG. (A) Specificity of LamR181-230 IgG using Western Blot analyses performed on E3.5 chicken embryo. **(B, C)** To identify the intracellular and cell membranous location of LamR, DRG were cultured on coverslips coated with PDL and laminin-1 for 48 hours and then, after fixation, were stained under permeabilizing **(B)** or non-permeabilizing conditions **(C)**, respectively. For permeabilized conditions, DRG were stained with cell nuclei-specific dye Hoechst 33342, NF-m (neurite marker), SOX10 (SCPs marker), and fluorescently conjugated phalloidin to detect F-actin. In non-permeabilizing conditions, DRG were stained with antibodies directed against LamR$_{181\text{-}230}$ or β1 integrin, phalloidin bound to a fluorescent dye (detection of F-actin), and Hoechst 33342 dye (staining of cell nuclei). Each color channel is presented as a grayscale.

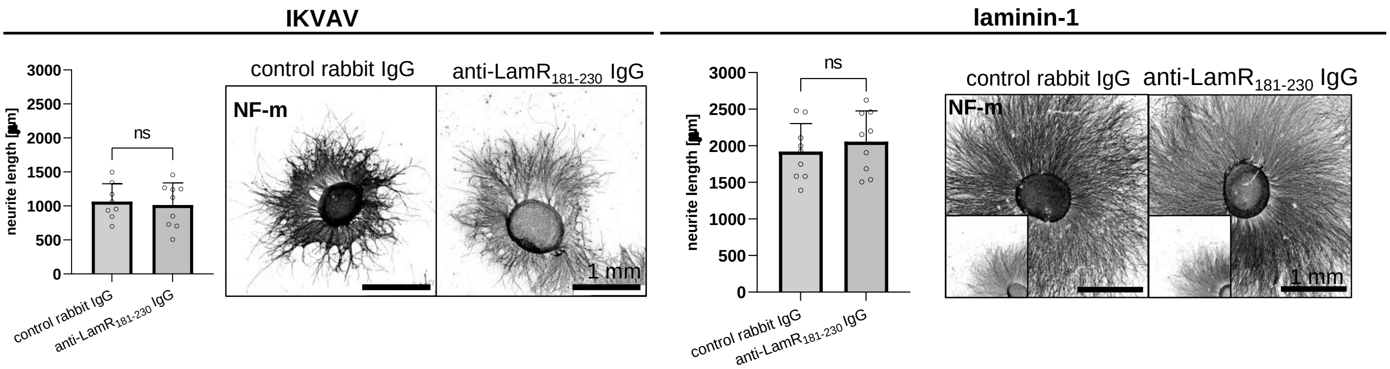

Supplementary Figure 5. Axonal outgrowth upon administration of anti-LamR$_{181\text{-}230}$ IgG. DRG were cultured on a PDL-coated surface in the presence of IKVAV peptide or laminin-1 for 48 hours. Antibodies directed against LamR$_{181\text{-}230}$ were also added to the medium to disrupt the biological activity of LamR. Control rabbit IgG were used in control conditions. Analysis of axonal outgrowth was done based on NF-m staining. The images were modified so that the black color represents the signal coming from the detected NF-m protein. The analysis was performed using the *NeuronJ* plug-in for ImageJ. Data represents mean ± SD. Data points represent the average length of the ten longest axons per DRG. $p \geq 0.05$ (non-significant; ns); unpaired t-test; (n = 9).

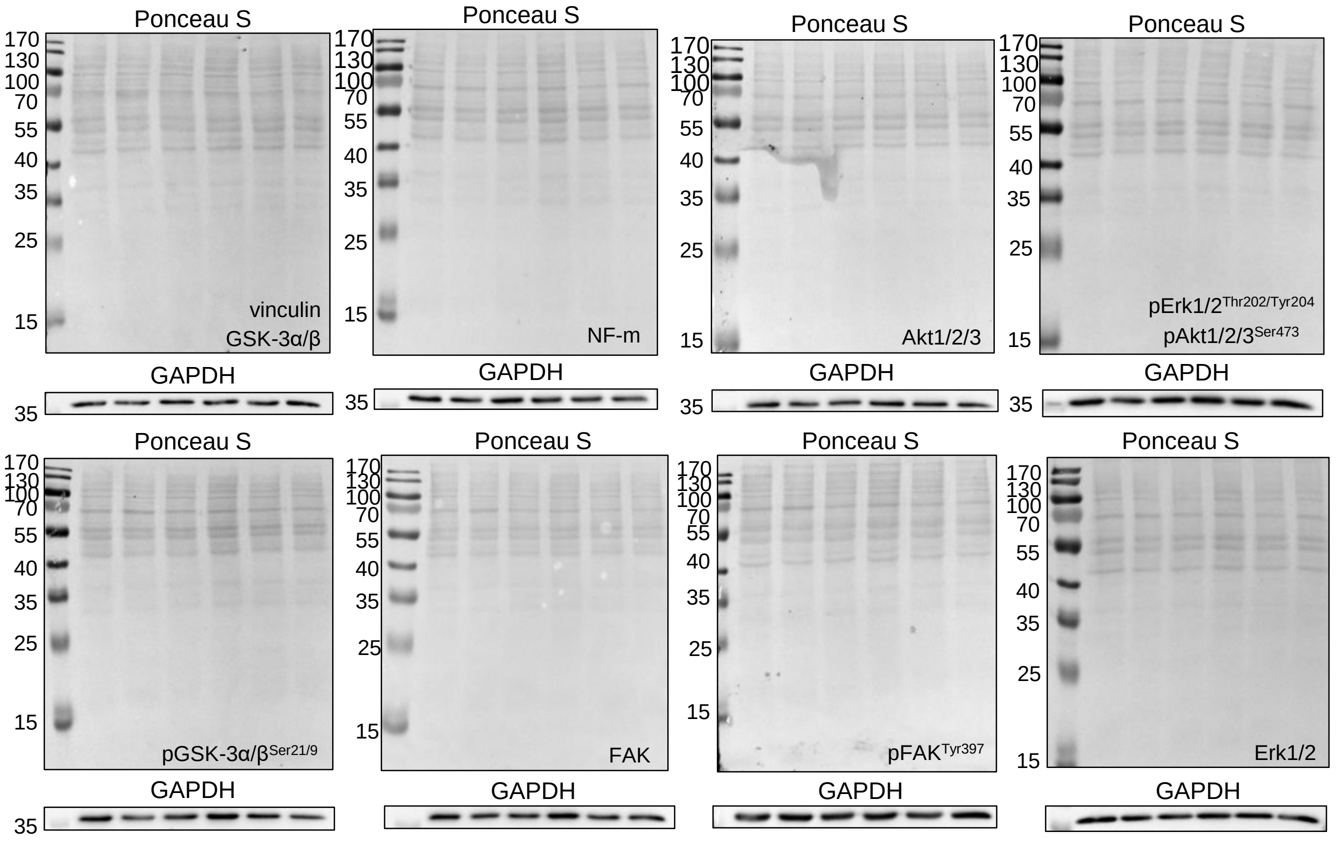

Supplementary Figure 6. Loading controls for membranes, which are shown in Figures 3 and 6. Membranes stained with Ponceau S and immunoblots with detected GAPDH. Each presented here pictures of membranes includes the name of protein detected on them.

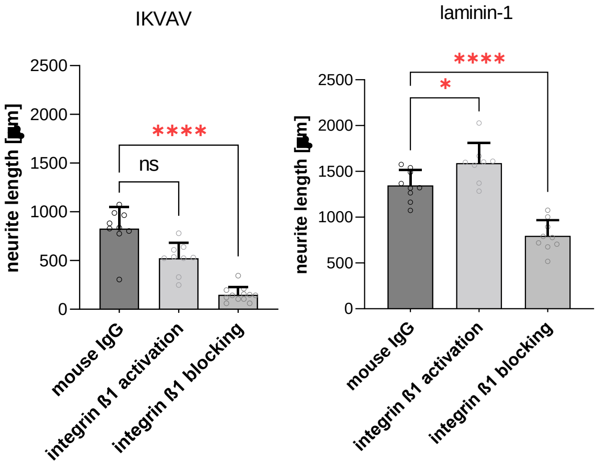

Supplementary Figure 7. The impact of integrin-directed IgG on β1 integrin activity. To activate β1 integrin, TASC/9D11 antibodies were used, whereas CSAT IgG were used to inhibit β1 integrin activity. Data points represent the average length of ten of the longest neurites per DRG. Data are shown as mean ± SD; $p \geq 0.05$ (non-significant; ns); $p < 0.05$ (*); $p < 0.01$ (**); $p < 0.0001$ (****); one-way ANOVA with Dunn's and Šídák's test; ($n = 9$).

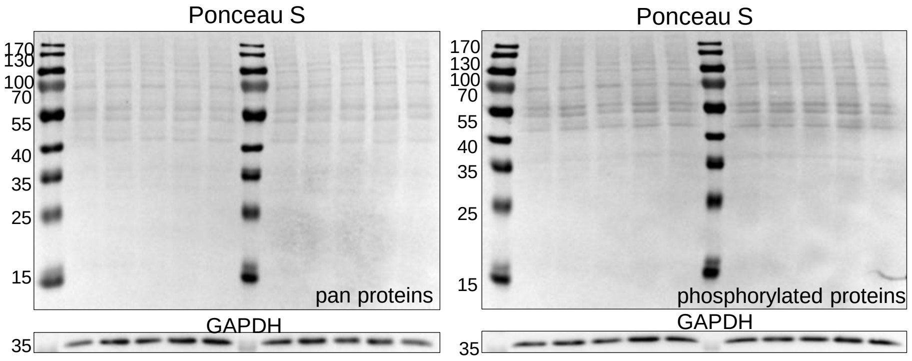

Supplementary Figure 8. Loading controls for analyzed membranes shown in Figure 7. Membranes stained with Ponceau S and immunoblots with detected GAPDH. Each presented here pictures of membranes includes the name of protein detected on them.

Supplementary Table 1. List of antibodies used in Western Blot analyses.

Name (clone)	dilution	Cat. Number.	Producer
**Primary antibodies**			
GAPDH 0411)	1:200	sc-47724	Santa Cruz Biotechnology
NF-m (4H6)	1:150	4H6	Developmental Studies Hybridoma Bank – DSHB
Vinculin (V284)	1:1000	MCA4656A	Bio-Rad
LamR	1:1000	PA5-86634	Thermo Fisher Scientific
LamR (A-7)	1:200	sc-376295	Santa Cruz Biotechnology
Akt1/2/3 (H-136)	1:200	sc-8312	Santa Cruz Biotechnology
pAkt1/2/3^{Ser473}	1:1000	9271S	Cell Signaling Technology
GSK-3α/β (0011-A)	1:200	sc-7291	Santa Cruz Biotechnology
pGSK-3α/β$^{Ser21/9}$ (D17D2)	1:1000	8566	Cell Signaling Technology
pFAK^{Tyr397}(D20B1)	1:500	8556	Cell Signaling Technology
FAK (C-903)	1:200	Sc-932	Cell Signaling Technology
pErk1/2$^{Thr202/Tyr204}$	1:1000	9102S	Cell Signaling Technology
Erk1/2	1:500	GTX134462	GeneTex
**Secondary antibodies**			
anti-mouse HRP-conjugated IgG	1:4000	7076	Cell Signaling Technology
anti-rabbit HRP-conjugated IgG	1:4000	7074	Cell Signaling Technology
